# Acute endothelial stresses identify microRNA let-7b-5p and non-coding *SLC11A2 (NRAMP2/DMT1)* exon as biomarkers that overlap with those detected in malignant and non-malignant diseases

**DOI:** 10.1093/qjmed/hcae235

**Published:** 2024-12-10

**Authors:** Adrianna M Bielowka, Dilip Patel, Dongyang Li, Maria E Bernabeu-Herrero, Laurence Game, Micheala A Aldred, Inês G Mollet, Claire L Shovlin

**Affiliations:** National Heart and Lung Institute, Imperial College London, London, UK; NIHR Imperial Biomedical Research Centre, London, UK; National Heart and Lung Institute, Imperial College London, London, UK; NIHR Imperial Biomedical Research Centre, London, UK; National Heart and Lung Institute, Imperial College London, London, UK; NIHR Imperial Biomedical Research Centre, London, UK; National Heart and Lung Institute, Imperial College London, London, UK; NIHR Imperial Biomedical Research Centre, London, UK; Genomics Facility, UKRI MRC Laboratory of Medical Sciences, London, UK; Division of Pulmonary, Critical Care, Sleep, and Occupational Medicine, Indiana University School of Medicine, Indianapolis, USA; UCIBIO-Requimte, Faculdade de Ciências e Tecnologia (FCT), Universidade NOVA de Lisboa, Caparica, Portugal; National Heart and Lung Institute, Imperial College London, London, UK; NIHR Imperial Biomedical Research Centre, London, UK; Respiratory Medicine, Imperial College Healthcare NHS Trust, London, UK

## Abstract

**Background:**

Disease biomarkers are often identified long after initiating pathologies, hampering mechanistic understanding and the development of preventative strategies. We hypothesized that aberrant cellular responses to normally-encountered stresses may be relevant to later disease states.

**Aim:**

To model two under-explored acute cellular stresses for blood-exposed cells, and cross-reference to known biomarkers of disease.

**Methods:**

Normal primary human endothelial cells (ECs) were treated for 1–6 h with cycloheximide (CHX) 100 μg/ml to inhibit protein translation and nonsense-mediated decay (modelling the integrated stress response), or 10 μmol/l ferric citrate (modelling diurnal variation in serum iron that can be augmented by treatments prescribed 8 million times/year in England). Directional whole transcriptome RNA-seq identified differentially expressed genes and micro(mi)RNAs. Customized novel scripts examined the expression of 517 225 exons to predict 1 h CHX-stabilized exons. Validations were by cel-miR-39-spiked qRT-PCR and RNA-seq in other EC types, peripheral blood mononuclear cells (PBMCs) and plasma.

**Results:**

miRNAs fell transiently at 1 h after 10 μmol/l ferric citrate (*P* < 0.01), specifically in let-7 family member pre-miRNAs (‘let-7’, *P* < 0.05), where there was an accompanying differential 6 h increased expression of 570 let-7-target mRNAs identified through TargetScan (*P* < 0.0001). qRT-PCR and RNA-seq validations in other normal ECs, plasma and PBMCs confirmed up to 80% falls in pre-let-7b/let-7b-5p after 1 h iron, and exon 3B of the *SLC11A2* (*NRAMP2/DMT1*)-encoded iron/copper transporter as a novel exon most consistently stabilized following 1 h treatment with CHX. Overlaps with disease biomarkers for cancer, growth retardation and multiple organ-specific diseases were identified.

**Conclusions:**

Biomarkers for normal, acute cellular responses overlap with disease-state biomarkers, warranting further study.

## Graphical abstract

**Figure:**
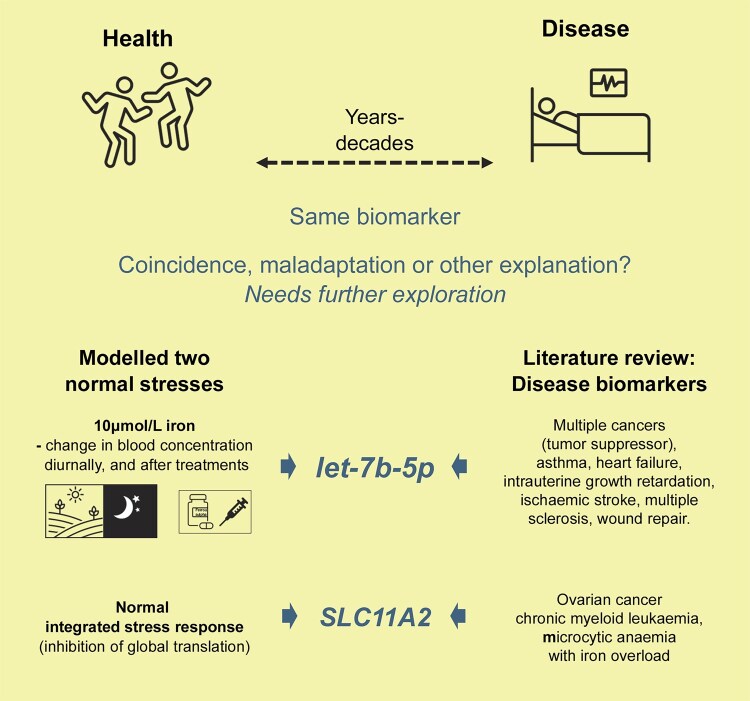

## Introduction

Disease pathogenesis can span decades, as exemplified by cancer latency periods after mutagen exposure, and the lag between inhalation of toxic agents to the development of pulmonary fibrosis. Where causative pathogenesis occurs long before the development of disease recognition, it is challenging to define the full biological relevance of biomarkers identified many years later.^1^ Based on clinical observations in an inherited vascular disorder,^2^ we hypothesized that acute cellular responses to physiologically encountered stresses may play roles in longer-term disease pathogenesis. Eukaryotic organisms have evolved to minimize damage by activating specific RNA and protein responses within minutes of any injury, facilitating restoration of cellular health where feasible and limiting future damage by promoting adaptation to environmental stimuli.^3^ We reasoned that since vascular endothelial cells (ECs) are exposed to fluctuating blood-borne injurious agents, failure to respond in appropriate ways may lead to subsequent pathology. ECs are the driver cells for cardiovascular human disease, including atherosclerosis, a process that evolves over decades. Further, ECs are increasingly recognized to support the health of adjacent tissues, for instance at endothelial-epithelial interfaces in the lung.^4^

**Figure 1.**
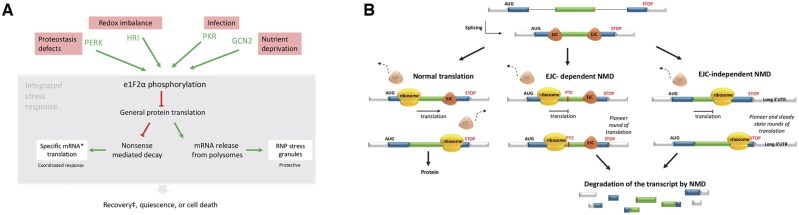
The integrated stress response (ISR) and NMD. For further details regarding these stylized representations, the reader is referred to recent reviews.^5^^,^^6^^,^^9^ (**A**) Overview of the ISR. In unstressed cells, translation initiation proceeds because the alpha subunit of eukaryotic initiation factor 2 (eIF2α) is maintained in an unphosphorylated form, facilitating assembly of the charged methionyl-initiator tRNA eIF2•GTP•methionyl-intiator tRNA ternary complex. In contrast, in stressed cells, E1F2α is phosphorylated at serine 51 which prevents eIF2β GDP-GTP exchange. Stress-specific kinases phosphorylate eIF2α (PERK, protein kinase RNA-like ER kinase; HRI, heme-regulated eIF2α kinase; PKR, protein kinase R; GCN2, general control nonderepressible 2 kinase). Phosphorylation enables eIF2α reprogramming of gene expression to maintain cellular viability while responding to injury. *Specific mRNAs where translation activates expression of genes involved in cellular adaptation include ATF3, ATF4, ATF5, CEBPA, CEBPB and CHOP. RNP, ribonucleoprotein (protein-associated RNA).‡The ISR is terminated by dephosphorylation of phosphorylated eIF2α by protein phosphatase 1 (PP1) and its regulatory subunits GADD34 (growth arrest and DNA damage-inducible protein 34) and CReP (constitutive repressor of eIF2α phosphorylation).^6^ If the injury/stress cannot be overcome, apoptosis is triggered.^5^^,^^6^ (**B**) Overview of NMD which is inhibited during the ISR. NMD predominantly takes place while a newly synthesized and spliced mRNA remains bound by the CBP20-CBP80 cap-binding protein (CBP) heterodimer, during the first (‘pioneer’) round of protein translation. Exon junction complexes (EJCs) comprising multiple splicing factors, mRNA export proteins and core EJC components, are deposited upstream of the exon–exon junctions during pre-mRNA splicing,^9^ and are displaced as the ribosome moves along the transcript during the first round oftranslation. Since natural (‘physiological’) stop codons are usually located in the terminal exon, once protein translation is completed, all EJCs will have been removed. However, if a PTC is present at least 50–55 nucleotides upstream of the last exon-exon boundary, on translation terminatation at the PTC, at least one EJC will remain bound to the mRNA, thus triggering the transcript for NMD. Other references for NMD/NMD escape are provided elsewhere.^15^

We examined two stresses relatively unexplored for human disease. The first was a model of the integrated stress response, when cells respond not only by activation of specific survival programmes, but also by transient inhibition of global protein synthesis.^5^ Global protein translation inhibition results when EIF2α is phosphorylated by kinases that sense diverse cytosolic stresses and is terminated by EIF2α dephosphorylation (Figure 1A).^5^^,^^6^ Experimentally, protein translation can be directly inhibited in otherwise unstressed cells by cycloheximide (CHX) which binds to the 60S ribosomal subunit E-site.^7^ Secondary consequences of global translation inhibition include attenuation of nonsense-mediated decay (NMD)^8^ which is one of the major physiological processes that promotes translational fidelity (Figure 1B).^9^ Thus, examining cellular responses to CHX would provide a window on a recurring state that cells need to adapt to in order to survive,^10^ and potentially new biomarkers for diseases triggered by diverse cellular stresses.

The second stress for which we sought endothelial biomarkers was a redox challenge which results from changes in extracellular iron concentrations. Diurnal changes in total serum iron have been shown to average 12 μmol/l over 24 h,^11^ and there are similar magnitude changes in non-transferrin bound iron in a proportion of individuals following oral or intravenous iron treatments.^12^^,^^13^ Iron tablets are prescribed over 8 million times a year in England to treat iron-deficiency anaemias,^14^ and are also available for purchase without prescription from pharmacies, supermarkets and online. We had been alerted to possible acute endothelial consequences by patients who commonly require iron treatments for anaemia, and are also able to recognize and report vascular changes in real time due to nosebleeds.^2^^,^^15^ Across two separate, unbiassed surveys of iron-using patients with hereditary haemorrhagic telangiectasia (HHT), a total of 53 of 1049 (5.1%) iron tablet users, and 33 of 398 (8.3%) iron infusion users reported that these treatments, but not control investigations/treatments, exacerbated their nosebleeds.^16^^,^^17^ In healthy volunteers ingesting a single ferrous sulphate 200 mg tablet, we demonstrated transient rises in circulating endothelial cells (cECs, viable CD34^+^CD45^−^CD146^+^ cells^16^) which were an accepted marker of endothelial damage,^18^ and are now also recognized as displaying markers of EC senescence.^19^ We also reported that treatment of normal human primary ECs with low dose iron directly induces a DNA damage response.^20^

Here, we report novel biomarkers for these acute responses and overlaps with known disease biomarkers.

## Materials and methods

Further detail is provided in the Supplementary Data.

### Treatment optimizations in endothelial cells

Since prolonged CHX and high-dose ferric citrate treatment results in EC death, treatment concentrations were optimized in ECs during pilot experiments. Final studies used CHX (Sigma-Aldrich, USA) 100 μg/ml, a concentration already used to inhibit NMD,^8^^,^^21^ and ferric citrate (Sigma-Aldrich, USA) 10 μmol/l.

For treatment duration, no morphological changes were observed in primary human pulmonary microvascular ECs (HPMEC) or primary human dermal microvascular ECs (HDMEC, PromoCell GmbH, Heidelberg) cultured for 24 h with 10 μmol/l ferric citrate: 1 h and 6 h responses were evaluated. Minor morphological changes were observed after 3 h 100 μg/ml CHX treatment of HPMEC, but not HDMEC: CHX was tested for 1 h in HDMEC as a more resilient primary EC type, to minimize potential confounding due to later initiation of secondary and apoptotic pathways.

### RNA-sequencing (RNA-seq) and validations

Initial RNA-seq examined RNA from normal HDMEC and HPMEC (1 h treatments in HDMEC from one donor, 6 h treatments in HPMEC from a different donor). Ribosomal (r)-RNA-depleted total RNA was used to prepare strand-specific whole transcriptome libraries, and clusters generated for short-read sequencing on an Illumina platform. Valid reads were aligned to spliced transcripts from ExonMine^22^ using Seqmap.^23^ Differences in gene expression between two samples were evaluated using read counts for all exons and junctions for the gene strand only. Sequenced reads aligning to microRNA stem-loop sequences from miRBase were counted using custom Perl scripts, and normalized to the total number of valid reads and exon size. Scripts were written to select pre-miRNAs that demonstrated alignments that differed by at least 20% in treated cells. Binary sequence alignment map (bam) files were also analyzed in the Integrated Genome Browser (IGB) 9.1.8.^24^ Additional RNA-seq examined normal blood outgrowth ECs (BOECs) and peripheral blood mononuclear cells (PBMCs) isolated from healthy donors (four BOEC rRNA-depleted libraries,^15^ four BOEC size-fractionated, small RNA libraries, and six PBMC rRNA-depleted libraries,^25^ as described elsewhere).^15^^,^^25^^,^^26^

Genes demonstrating differential alignments were clustered using the Database for Annotation, Visualization and Integrated Discovery (DAVID).^27^ miRNA target genes were identified by TargetScan.^28^ Pre-miRNAs can give rise to two mature forms of miRNAs: mRNA targets of both 5′ (5p) and 3′ (3p) mature miRNAs were determined, and up to 3000 targets used for pathways analyses. For qRT-PCR validations, we spiked known concentrations of cel-miR-39 (Qiagen, USA) into 0.5 ml TRI preparations of cellular extracts before RNA extraction. For exon analyses, to increase power, RNA-seq data were also examined for six patients with HHT.^15^^,^^25^

### Statistical analysis

Creation of customized scripts and data analysis was performed in R, with additional analyses in STATA IC v15.0 (STATACORP, Texas, USA) and GraphPad Prism 9 (GraphPad Software, San Diego, CA, USA).

## Results

### Biological processes identified by mRNAs differentially expressed in normal EC after 1 h protein translation inhibition or 10 μM iron exposure

In HDMEC, after 1 h CHX treatment, genes encoding proteins involved in cancer pathways, protein degradation and helicase terms were strongly represented in the top 100 differentially expressed genes (Table S1Ai), particularly when clustering genes that were also differentially expressed in other experimental evaluations of NMD inhibition (Table S1Aii). Clusters of genes differentially expressed after 1 h 10 μmol/l ferric citrate were different (Table S1Bi), though similarities did emerge for genes differentially expressed after 6 h ferric citrate (Table S1Bii). These data supported examining the datasets in parallel, and considering CHX treatment as a model for NMD inhibition.

### Differentially expressed miRNAs in normal EC after 1 h protein translation inhibition or 10 μM iron exposure

In HDMEC, there were alignments to 113 pre-miRNAs, and for 34 (30%), expression differed by at least 20% (13 were higher in media-treated HDMECs, 21 higher in CHX-treated HDMEC). As shown in Figure S1, nearly one-third (31.6%) of miRNA target genes encoded proteins involved in the regulation of phosphorylation, one-sixth (13%) in phosphatidylinositol signalling, one-sixth (13%) in transcription and one-twentieth (5.2%) in cell adhesion.

A different picture emerged examining HDMECs treated with 10 μmol/l ferric citrate for 1 h. Although 13 of the 20 most highly expressed pre-miRNAs in media-treated HDMECs differed by at least 20% in iron-treated HDMEC, only one differed in the same direction as in the 1 h CHX-treated HDMEC and 4/20 (25%) differed in opposite directions.

### Differentially expressed miRNAs after 1 and 6 h exposure of normal EC to 10 μM iron

**Figure 2.**
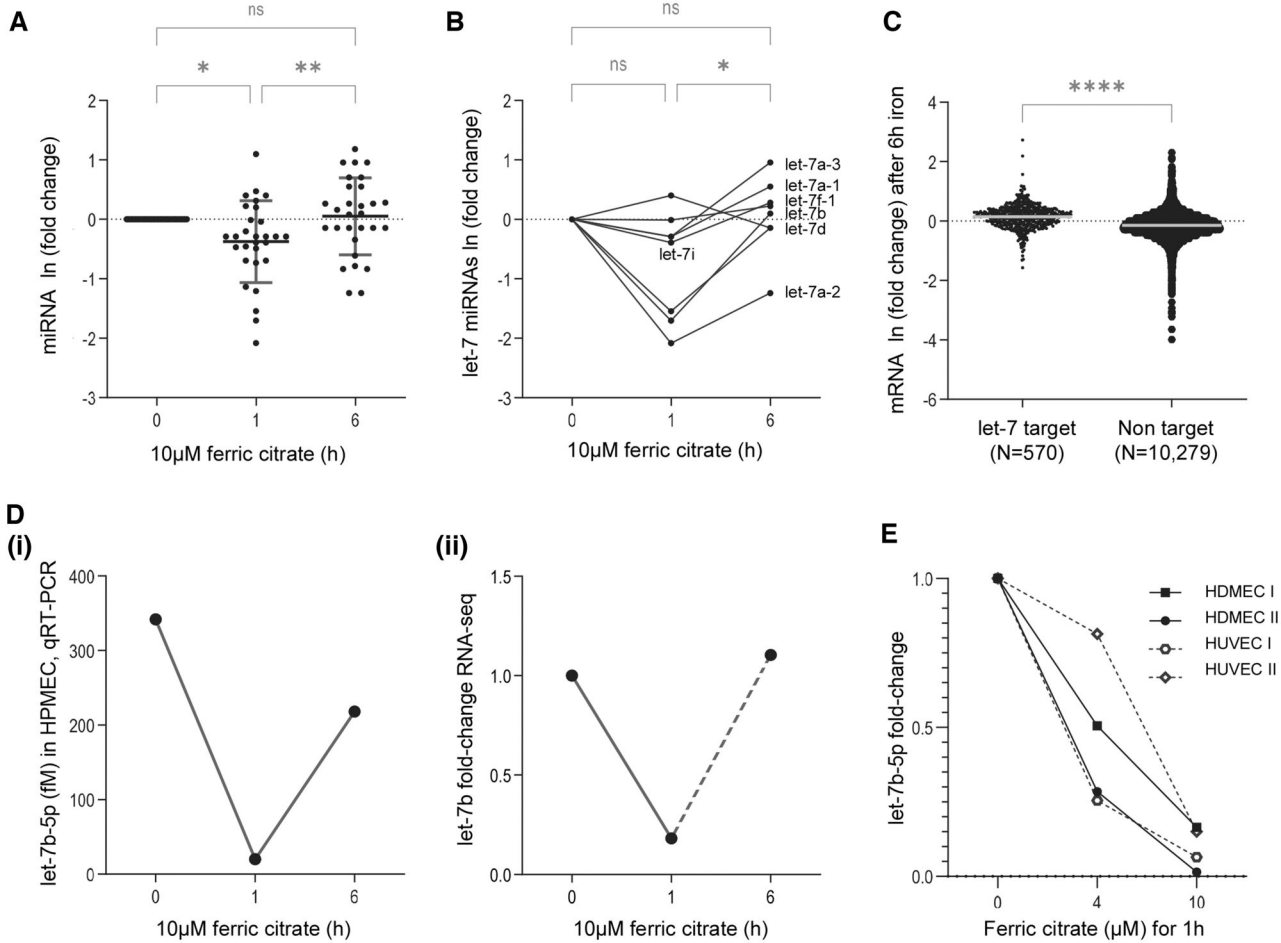
Pre-miRNA and mature miR responses to ferric citrate treatment of normal primary ECs. (**A**) RNA-seq data for all pre-miRNAs detected in 1 and 6 h rRNA-depleted libraries from EC treated with media or 10 μmol/l ferric citrate, presented as fold change of respective media-treated EC represented at 0 h. One hour data are from normal HDMEC compared to paired 1 h media-treated HDMEC. Six hour data are from normal HPMEC compared to paired 6 h media-treated HPMEC. By Dunn’s test post Kruskal–Wallis, there was a fall (* *P* < 0.05) after 1 h treatment, and no significant change after 6 h. (**B**) RNA-seq data for let-7 pre-miRNAs as in (A) (let-7a-1, let-7a-2, let-7a-3, let-7b, let-7d, let-7f-1, let-7g, let-7i), with *P* values (*P < 0.05) calculated by Dunn’s test post Friedman. (**C**) RNA-seq data for mRNA changes in HPMEC after 6 h 10μmol/l ferric citrate, as fold change of respective values in paired media-treated HPMEC, categorized by whether mRNAs were identified by TargetScan 5.2[28] as a let-7 target based on mature miRNAs from miRbase: 570 of 10 851 mRNAs had 1-6 (mean 1.11) let-7 family 8mer, 7mer-m8 or 7mer-1A binding sites (Table S2). (**D**) Comparisons of let-7b by qRT-PCR and RNA-seq. (i) Mature miRNA let-7b-5p, assayed at stated durations of 10μmol/l ferric citrate as quantified in HPMEC by qRT-PCR using Applied Biosystems miRNA assay 002619. Ct values were converted to concentrations following spiking of cell extracts with known concentrations of cel-miR-39 as described in the Supplementary Data. (ii) RNA-seq comparisons (as also represented in A and B). (**E**) Dose–response curves for mature let-7b-5p in four cultures of primary HDMEC or primary HUVEC, after 1 h treatment with media (‘0’), 4 or 10 μmol/l ferric citrate treatments, quantified by qRT-PCR using miRNA assay 002619 (Applied Biosystems). Threshold cycles (Ct) values were converted to concentrations based on known concentrations of spiked cel-miR-39 (described further in Supplementary Data), before fold-changes from the media-treated EC were calculated for graphical presentation.

Since EC viability meant it was feasible to concurrently evaluate pre-miRNAs at 1 h and target mRNAs at 6 h, we examined miRNA responses to iron treatment more closely. Alignments to the 29 pre-miRNAs expressed in all HDMEC and HPMEC datasets were lower in EC treated with 10 μmol/l ferric citrate for 1 h compared to media treatment, but no different between media and 10 μmol/l ferric citrate-treated EC at 6 h (Figure 2A). Since iron handling is a core requirement for all organisms, we reasoned that adaptive responses would involve the oldest miRNA families.^29^ Multiple let-7 family members (but not miR-100 or miR-125) were highly expressed in HDMEC and HPMEC. Let-7 miRNAs reproduced the overall pre-miRNA findings indicating a fall after 1 h iron treatment, and recovery by 6 h (Figure 2B). To test if there were functional consequences, we compared 6 h post-iron expression changes of 10 849 mRNAs in HPMEC, categorized by whether they were designated let-7 target mRNAs by TargetScan (Table S2). There was a significant increase in let-7 target mRNAs after 6 h treatment with 10 μM ferric citrate compared to mRNAs that were not let-7 targets (Figure 2C).

To validate miRNA changes by qRT-PCR, we tested normal HPMEC, HDMEC and human umbilical vein EC (HUVEC) where EC were ‘spiked’ with known concentrations of *C.elegans* miR-39 (cel-miR-39). Transient 1 h falls were confirmed for mature let-7 family members by qRT-PCR, and also for mature miR-21, miR-30 and miR-221 though their target mRNAs did not increase at 6 h. For the let-7 family pre-miR displaying the most substantial change at 1 h (let-7b), respective concentrations of mature let-7b-5p by qRT-PCR of HPMEC across 0-1-6 h (Figure 2Di), paralleled the RNA-seq data that had aggregated HPMEC 0-6 h with 0-1 h in HDMEC (Figure 2Dii). Examining further normal donors and EC types indicated that a 1 h fall in let-7b-5p was also evident following treatment with only 4 μmol/l ferric citrate (Figure 2E).

Next, we tested expression in samples more appropriate to wider population studies. Basal let-7b-5p expression was similar in PBMCs cultured for 1 h in native plasma at 37°C (fM:μ115; σ55) to ECs (fM:μ72; σ65). Furthermore, let-7b-5p changes in PBMC after 1–6 h 10 μmol/l ferric citrate were similar to HUVEC after 1–6 h 10 μmol/l ferric citrate (Figure S2). Notably, basal expression of let-7b-5p was ∼300-fold lower in plasma samples (fM:μ0.35; σ0.28) than in paired PBMCs (*P* = 0.0074).

**Figure 3.**
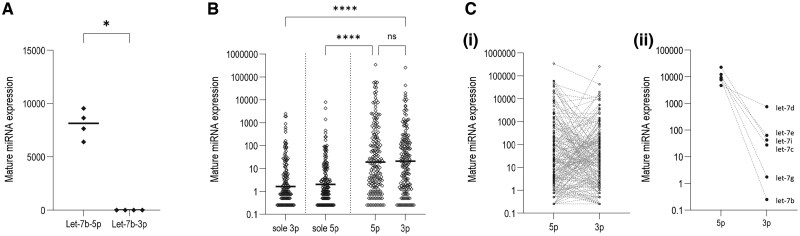
Small RNA-seq for mature miRNAs in normal BOECs. (**A**) Comparison of unique exact matches to let-7b-5p and let-7b-3p in four normal BOEC cultures. *P < 0.05: *P* = 0.029 by Mann–Whitney (and by Dunn’s post test following inclusion of the other datasets shown in Table S3). (**B**) Exact unique expression values for 458 miRNAs with unique, exact matches to mature 5p or 3p mature miRNAs, categorized by the 134 miRNAs (29%) where there were only alignments to the 3p strand (‘sole 3p’); 145 (32%) %) where there were only alignments to the 5p strand (‘sole 5p’); and 179 (39%) %) where there were alignments to both strands. ****P < 0.0001, calculated by Dunn’s test post Kruskal–Wallis. (**C**) Relationship between 5p and 3p strand expression for (i) all 179 miRNAs with both 5p and 3p strand expression, and (ii) let-7 miRNAs. Note let-7b demonstrated the greatest 5p strand bias within the let-7 family in these resting, normal BOEC.

Finally, to confirm mature miRNAs qRT-PCR assays had quantified all relevant mature let-7b species, we examined small RNA-seq data from four libraries of normal BOEC cultures (Table S3). It is known that there can be marked 5p or 3p strand biases for miRNAs, and this was evident for let-7b. As shown in Figure 3, let-7b-5p was expressed 8058-fold higher than let-7b-3p, with only a single let-7b-3p read across four separate cultures/libraries. The data suggested a 1 h fall in pre-let-7b effectively implied a fall in mature let-7b-5p. We concluded that let-7b (let-7b-5p) was an endothelial, PBMC and blood biomarker for an acute and functional response to modest increases in extracellular iron concentrations.

### Exon biomarkers for 1 h protein translation inhibition

**Figure 4.**
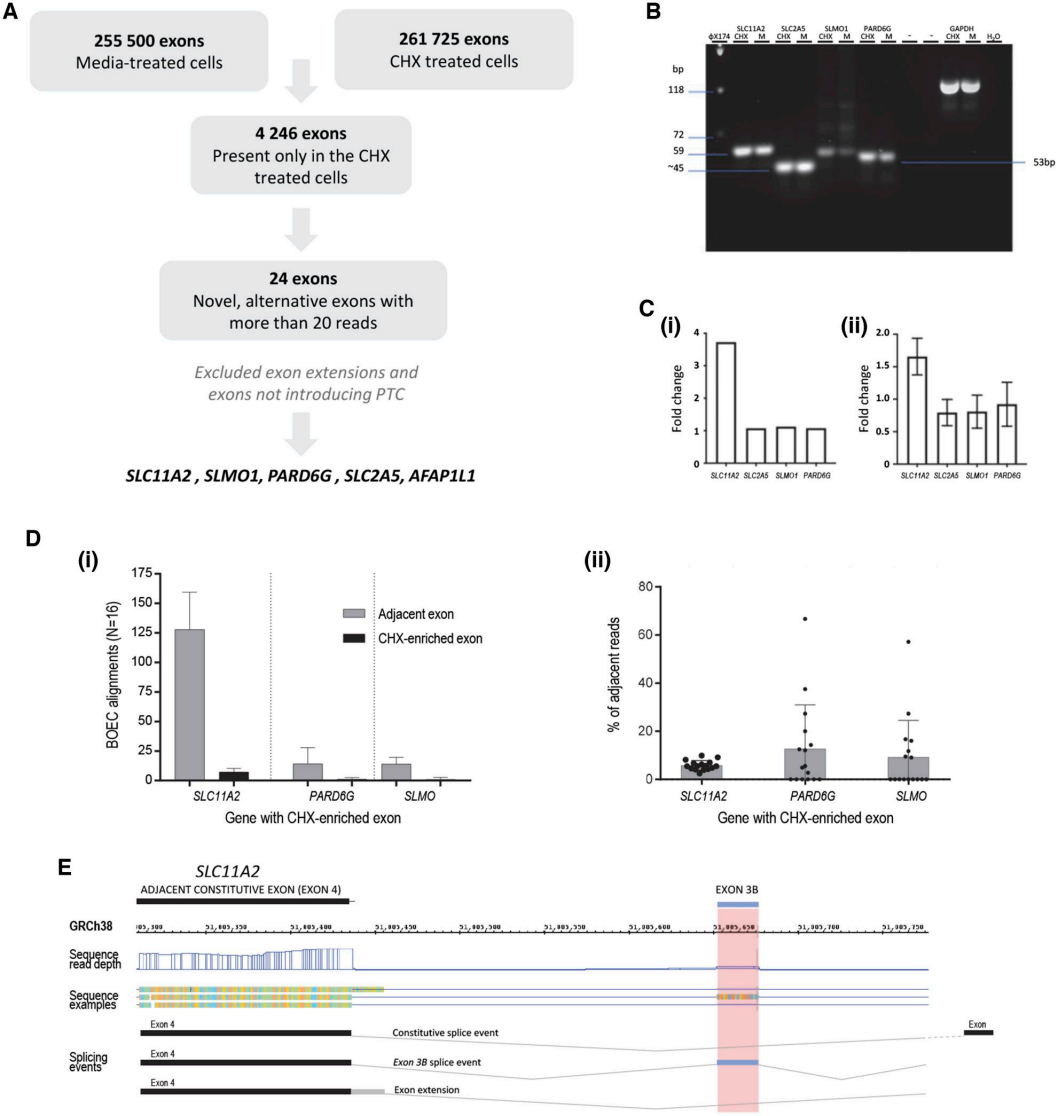
Exon biomarker for translation inhibition in normal ECs. (**A**) Selection of exons likely to be targeted by NMD which requires protein translation. Data from paired media- and CHX-treated EC were compared to identify exons where alignments were only identified in CHX-treated EC, and were to novel, alternative exons that introduce a PTC^9^^,^^10^^,^^15^ into the RNA transcript (details in Table S4). (**B**) RT-PCR of exons amplified in HUVEC treated for 1 h with 100 µg/ml CHX or fresh media, amplified using primers designed for exons likely targeted by NMD (Table S4). Products were size-separated by low melting agarose gel. Abbreviations: CHX, cycloheximide, H_2_O, water, bp, base pairs. (**C**) Fold change of candidate exons in ECs treated for 1 h with 100 µg/ml CHX compared to fresh media: (i) HUVEC and (ii) BOECs. Data from *AFAP1L* not shown as this demonstrated low level of amplification using all tested primers. (**D**) Alignments to the CHX-enriched exon, and nearest adjacent exon for three separate genes, across 16 BOEC cultures from four separate donors including three with HHT^25^ to enhance statistical power. Mean and standard deviation displayed for (i) absolute alignments and (ii) percentage of alignments to the nearest adjacent exon. (**E**) Integrated Genome Browser (IGB) 9.1.8 views of GRCh38 chr12:51,005,295-51,005,797 spanning the CHX-enriched exon 3B and adjacent constitutive exon 4 of *SLC11A2*, showing reads from a single normal BOEC library. The position of exon 3B is highlighted across GRCh38 coordinates. Note the sharp boundaries of alignments to exon 3B, and its absence from the RefSeq curated transcript denoted by a constitutive splicing cartoon.

To identify novel endothelial biomarkers for protein translation inhibition, we turned to specific exons. There were 255 500 alignments to individual exons in the media-treated HDMEC and 261 725 alignments in 1 h CHX-treated HDMEC, with 4246 exons observed only in the CHX-treated cells. After applying the criteria for selection of novel, alternative exons with more than 20 reads, the list was reduced to 24 exons (Table S4), then a final prioritized list that comprised 5 exons (Figure 4A).

Quantitative (q)RT-PCR validations were performed on cDNA from HUVEC (Figure 4B, Figure 4Ci), and BOECs (Figure 4Cii). In crude and GAPDH-normalized analyses, expression of the *SLC11A2* 3B exon emerged as consistently higher in all EC types examined after treatment with CHX, with an approximately 4-fold increment in CHX-treated HUVEC (Figure 4Ci) and a 1.7-fold increase in BOECs (Figure 4Cii).

Binary alignment map (bam) files demonstrated that for *SLC11A2, PARD6G* and *SLMO1*, the exon sequences were expressed in a small proportion of normal, untreated primary BOECs (Figure 4Di). *SLC11A2* exon 3B was expressed at a consistently low level compared to the adjacent exon (mean 5.9%, SD 2.0%), with more variable expression of the *PARD6G* and *SLMO* candidate exons (Figure 4Dii). We reasoned that if exons are solely NMD targets unmasked by CHX,^8^^,^^9^ then the majority of RNA-seq alignments should be to fully spliced exons, and this was seen for *SLC11A2* exon 3B (Figure 4E). For *PARD6G* exon 2A and *SLM01* exon 1B, such precise splicing patterns were not observed (Figure S3). We concluded that the novel *SLC11A2* exon 3B was in a transcript most consistently stabilized by NMD inhibition, and with similar expression patterns in BOECs and PBMCs (Figure S4), suitable for biomarker development.

### Disease relevance of *SCL11A2*

*SLC11A2* (also known as *NRAMP2 or DMT1*) is expressed at modest levels (median 4.05–35.21 transcripts per million (TPM)) in bulk RNA-seq analyses across 53 tissues in the Genotype Tissue Expression Project (GTEx),^30^ and encodes an essential iron/copper transporter where common^31^ and rare^32^ variants result in microcytic anaemia with iron overload. More recently, *SLCA11A2* overexpression has been reported as a promising biomarker and therapeutic target in ovarian cancer,^33^ and chronic myeloid leukaemia.^34^ Alternate 5′ and 3′ splicing is known to critically regulate *SLCA11A2* subcellular location and site of iron transport,^35^^,^^36^ with alternate splice isoforms shown to act as binary switches controlling cell fate decisions in normal and tumour cells.^37^ In the course of this project, exon 3B was identified within a RefSeq non-coding RNA transcript, NR 183176.1 (*SLC11A2* transcript variant 25) originally from a Roswell Park Cancer Institute Human BAC Library.^38^  *SLC11A2* encodes a divalent metal transporter 1 (DMT1) responsible for absorption and transport of metals including cadmium, copper, zinc, iron and manganese, therefore inclusion in toxicity assays may prove valuable.

### Disease relevance of let-7b and let-7b-5p

The let7-b host gene (*MIRLET7BHG*) is expressed across the 53 GTEx tissues at median 0.17-6.23 TPM.^30^ Let-7 (*lethal-7*) miRNAs were the first described human microRNA family, originally identified due to essential developmental regulatory roles.^39^ Tightly regulated, the let-7 family have long-established identities as tumour suppressors,^40^^,^^41^ and are considered as potent therapeutic targets to inhibit tumorigenesis.^41^ Beyond cancer, let-7 family members are emerging as biomarkers for organ-specific diseases including asthma,^42^ myasthenia gravis^43^ and intra-uterine growth retardation^44^ with let-7b-5p specifically identified in models of neonatal encephalopathy.^45^ Let-7b-5p has also been identified as a biomarker for ischaemic stroke,^46^ and heart failure^47^ with potential proposed for both diagnostic and therapeutic strategies; and as a ‘meta-miRNA’ at the hub of a network of seven miRNAs tightly linked to multiple sclerosis.^48^ Notably, inhibition of Let-7b-5p maturation has been shown to be beneficial in promoting skin repair after burn injury.^49^

## Discussion

We have identified biomarkers for acute stress responses in normal human primary EC, demonstrated biomarker potential in blood/PBMCs, and reported relevance to disease states. We specifically showed that 1 h inhibition of protein translation (a model of the integrated stress response), induces the inclusion of an alternatively spliced exon from the *SLC11A2* (*NRAMP1/DMT1)* gene that encodes a divalent metal transporter, and also identified miRNA let-7b (let-7b-5p) as a biomarker for acute iron exposure responses in diverse EC types and circulating PBMCs.

Study strengths include the linking of acute cellular stresses to biomarkers that can be identified years later in well-established disease; exacting methodology including concurrent miRNA/mRNA evaluations in the same and serial datasets to allow target gene validations; cell extract spiking with cel-miR-39 to enable absolute concentration calculations; experimental validations across multiple EC types, PBMCs and plasma samples, and cross referencing to previously published data in disease-specific domains. We mitigated a potential study weakness (induction of cell death by prolonged CHX/iron treatments) by selecting cell types demonstrating greater resilience and restricting treatments to lesser durations such as a 1 h time-course that is also more analogous to physiological stresses than the more usually studied 24–72 h.^50^^,^^51^ A further study strength was the overlap in differentially expressed genes after CHX treatments of differentiated human cells, with genes differentially expressed in transformed cells and pluripotential cells from other species after targeting individual NMD factors.^50^^,^^51^

Weaknesses are that experimental numbers are modest; direct causal links are not established; and mechanisms require further study. Further, the mature miRNA RNA-Seq datasets are at baseline only. That said, the study provides new tools by which the causal links can be explored in experimental, clinical and population-level studies relevant to major population-level health insults.

One of our foci was to model the stress inhibition of NMD, one of the major physiological processes that promotes translational fidelity, and provides RNA-based responses to triggers that would otherwise perturb cellular homeostasis.^9^^,^^52^ Regulated unproductive splicing and translation (RUST) describes the regulation of gene expression through the coupling of NMD and alternative splicing,^53^ including the use of upstream open reading frames (uORFs) which contain a PTC, and multiple NMD roles are described in the regulation of gene expression.^9^^,^^50^^,^^51^^,^^54^^,^^55^ Additionally, NMD is considered a therapeutic target where aberrant premature termination codons (PTCs) are generated due to somatic or germline DNA sequence variants that cause disease,^56–58^ or for tumours that use NMD to downregulate tumour suppressor genes.^59^^,^^60^ Since inhibition of NMD is a nonspecific strategy, this would stabilize not only mRNAs with nonsense mutations, but also natural targets of NMD. Acute studies such as the current experiments, may therefore illuminate cellular adaptations including in stressed cells when NMD is suppressed, as discussed further elsewhere.^10^

Our second study focus was to model the serum iron changes that are observed in a proportion of iron users^12^^,^^13^^,^^61^^,^^62^ after conventional treatments (Table S5), noting these are superimposed on similar magnitude diurnal changes in serum iron.^11^ Iron deficiency is a global health priority, necessitating correction by iron supplements,^63^ and ferrous salts are a WHO Essential Medicine for tablets equivalent to 60 mg elemental iron, and oral liquids equivalent to 25 mg iron/ml.^64^ More recent understanding has allowed recognition that natural regulatory axes limit potentially deleterious effects of iron, while still delivering iron to target proteins, and reduce gastrointestinal tract absorption of iron from tablets containing >40 mg elemental iron.^65^^,^^66^ Indeed, the NIH-sponsored Trial to Assess Chelation Therapy TACT2^67^ did not replicate the cardiovascular event risk reduction from effective iron chelation seen in TACT1.^68^ Our study findings support a model where key mRNAs can be held in check by high steady-state levels of let-7b-5p that fall rapidly in response to iron exposure, derepressing translation of a subset of proteins to serve the adaptive response. miRNAs contribute directly to translational repression, and there is compelling evidence to suggest that target degradation of mRNAs is a major mode of miRNA- and particularly let-7b-mediated gene silencing.^39^ Our data suggest such repression would need to be very early, and support examination of earlier time courses for miRNA regulation than generally studied.

In conclusion, the study contributes to general understanding and provides tools for future studies that can now formally test pathology causation for the acute cellular stresses examined in the current study.

## Supplementary Material

hcae235_Supplementary_Data

## Data Availability

Non-sensitive data underlying this article are to be made available at Zenodo to be used under the Creative Commons Attribution licence. Primary sequence data used in this research was collected subject to the participants’ informed consent. Access to these data will only be granted in line with that consent, subject to approval by the project ethics board and under a Data Sharing Agreement. Blood outgrowth ECs used in this research were collected subject to the informed consent of the participants. Access will only be granted in line with that consent, subject to approval by the project ethics board.
