# Supplementary material for: Acute endothelial stresses identify microRNA let-7b-5p and non-coding SLC11A2 (NRAMP2/DMT1) exon as biomarkers that overlap with those detected in malignant and non-malignant diseases
Source: QJM. 2024 Dec 10;118(9):679–88. doi: 10.1093/qjmed/hcae235 (PMC12668437; doi:10.1093/qjmed/hcae235)
Supplement: hcae235_Supplementary_Data [file hcae235_supplementary_data.zip › hcae235_Supplementary_Data/AbbreviationsR1.2.docx]

**Abbreviations**

| bam | binary sequence alignment map files |
| --- | --- |
| BOEC | blood outgrowth endothelial cells |
| cECs | circulating endothelial cells quantified as viable CD34^+^CD45^−^CD146^+^ cells |
| CHX | cycloheximide |
| ECs | endothelial cells |
| h | hour |
| HDMEC | human dermal microvascular endothelial cells |
| HHT | hereditary haemorrhagic telangiectasia |
| HPMEC | human pulmonary microvascular endothelial cells |
| HUVEC | human umbilical vein endothelial cells |
| ISR | integrated stress response |
| let-7 | lethal 7 miRNA |
| let-7b-3p | mature let-7b 3p strand |
| let-7b-5p | mature let-7b 5p strand |
| miRNA | micro RNA |
| mRNA | messenger RNA |
| NMD | nonsense mediated decay |
| NTBI | non transferrin bound iron |
| PBMCs | peripheral blood mononuclear cells |
| pre-miRNAs | precursor microRNAs (hairpin doublestranded duplex after processing by Drosha) |
| qRT-PCR | quantitative reverse transcriptase polymerase chain reaction |
| RNA-seq | RNA-sequencing |
| RNP | ribonucleoprotein: RNA associated with protein |
| rRNA | ribosomal RNA |
| *SLC11A2* | also known as *NRAMP2* and *DMT1*: gene encoding solute carrier family 11 member 2 |
