# Supplementary material for: Acute endothelial stresses identify microRNA let-7b-5p and non-coding SLC11A2 (NRAMP2/DMT1) exon as biomarkers that overlap with those detected in malignant and non-malignant diseases
Source: QJM. 2024 Dec 10;118(9):679–88. doi: 10.1093/qjmed/hcae235 (PMC12668437; doi:10.1093/qjmed/hcae235)
Supplement: hcae235_Supplementary_Data [file hcae235_supplementary_data.zip › hcae235_Supplementary_Data/Bielowka et al R1 Data Supplement 2024.12.03.docx]

*TRANSLATIONAL SCIENCE*

**Acute endothelial stresses identify microRNA let-7b-5p and non-coding *SLC11A2 (NRAMP2/DMT1)* exon as biomarkers** **that overlap with those detected in malignant and non-malignant diseases**

Adrianna M. Bielowka, Dilip Patel, Dongyang Li, Maria E Bernabeu-Herrero, Laurence Game, Micheala A. Aldred, Inês G. Mollet, Claire L Shovlin

**DATA SUPPLEMENT:**

[SUPPLEMENTARY METHODS 2](#_Toc160393111)

[Cell culture 2](#_Toc160393112)

[Endothelial cell culture 2](#_Toc160393113)

[Endothelial cell treatments 2](#_Toc160393114)

[RNA preparations including cel-miR-39 and qRT-PCR 2](#_Toc160393115)

[RNA-seq 3](#_Toc160393118)

[Selection of cycloheximide (CHX)-enriched exons 3](#_Toc160393118)

[Peripheral blood mononuclear cell (PBMC) expression 3](#_Toc160393119)

[TABLES 4](#_Toc160393120)

[Table S1: Representative pathway terms identified after CHX or ferric citrate treatments. 4](#_Toc160393121)

[Table S2: (abridged): Details of let-7 target mRNAs in 6h data sets identified by TargetScan 6](#_Toc160393122)

[Table S3: Small RNA sequencing of mature miRNAs in endothelial cells (BOECs) 7](#_Toc160393122)

[Table S4: Novel alternate exons in CHX-treated HDMEC 8](#_Toc160393123)

[Table S5: Iron Prescription Data in England, financial year 2022/2023 9](#_Toc160393121)

[FIGURES 10](#_Toc160393124)

[Figure S1: Comparisons of media and CHX-treated HDMEC 10](#_Toc160393125)

[Figure S2: let-7b-5p expression in HDMECs, plasma and PBMCs 11](#_Toc160393126)

[Figure S3: Comparison of expression patterns of CHX-enriched exons in BOECs. 12](#_Toc160393126)

[Figure S4: *SLC11A2* expression in PBMCs and BOECs. 13](#_Toc160393126)

[SUPPLEMENTARY REFERENCES 14](#_Toc160393128)

**TABLE S2 (full)**

# SUPPLEMENTARY METHODS

## Cell culture

Endothelial cell culture
All cultures were of primary endothelial cells (ECs), cultured in antibiotic-free media. No endothelial cell lines were used.

Normal human dermal microvascular EC (HDMEC) and normal human pulmonary microvascular EC (HPMEC) were from PromoCell GmbH, Heidelberg where donors sign an informed consent form which details the purpose of the donation ([www.promocell.com/ethics](http://www.promocell.com/ethics)). HDMEC #0020208.1 had been isolated from the facial skin of a 63 year old white female, had a population doubling time of 26.6h, and 650,000 proliferating cells were reecived in passage 2. HPMEC #0032410.9 had been isolated from peripheral lung tissue of a 52 year old white male, had a population doubling time of 30.7h, and 600,000 proliferating cells were received in passage 2. EC were supplied as CD31+, VWF+, Dil-Ac-LDL+, smooth muscle actin negative, free of bacterial, fungal, mycoplasma, HIV-1 or HBV/HCV. On arrival, after 2h at 37°C, 5% CO_2_, media was replaced with fresh reconstituted Promocell microvascular EC media with 5% fetal calf serum. After 3 passages to optimise equivalence in replicate wells, EC were distributed to 6-well plates where wells were treated at confluence 7d (HPMEC) and 9d (HDMEC) after arrival. Experimental HDMEC/HPMEC RNA extractions and sequencing were performed in parallel.

Study of locally derived human umbilical vein EC (HUVEC) was approved by Hammersmith Hospitals Research Ethics Committee (Ref 06/Q0406/21). These were derived from separate donors, and cultured as described.[1] Human blood outgrowth endothelial cells (BOECs), were also studied as approved by East of Scotland Research Ethics Service (Ref 16/ES/0095), and cultured as described.[2] HUVEC and BOEC were passaged according to confluency, to final experimental treatment wells.

For all EC experimental wells, at confluence, media was replaced with fresh, prewarmed media with or without the relevant treatments (qv) for 1-6h. Immediately post-treatment, the adherent EC were washed *in situ* with fresh pre-warmed media before lysing in TRI reagent (Sigma, UK) at 0.3-0.4 mL per 1x10^5^-10^7^ cells), and immediate storage at -70^o^C.

### Endothelial cell treatments

Cycloheximide (CHX, Sigma Aldrich C1988, Gillingham, UK) was dissolved in molecular grade water to a stock concentration of 10mg/mL (100X), and frozen in aliquots at -20^o^C prior to use at final concentration 100µg/mL. Ferric citrate (Sigma Aldrich F3388, Gillingham, UK) was prepared each experimental day to stock concentration 10mmol/L (1000X), by dissolving in molecular grade water on a rocking platform for at least one hour at 56^o^C, before filter-sterilizing, as described,[3] and supplementing media to final concentrations of 4μmol/L and 10 μmol/L.

### RNA preparations including cel-miR-39, and qRT-PCR

For qRT-PCR validations, RNA was extracted as per TRI reagent manufacturer’s protocols using isopropyl alcohol/glycogen precipitation, a 75% ethanol wash, then air dry before resuspension in RNase-free water. For quantifications, TRI preparations were spiked with cel-miR-39 (Qiagen, US) prior to RNA extraction and qRT-PCR (Table SA1). Linear regression performed in STATA (Statacorp, Texas, US) enabled conversion of all threshold cycle (Ct) values to concentration values.

| **Cel-miR-39 nM** | **HUVEC Ct** | **HPMEC Ct** |
| --- | --- | --- |
| 20 |  | 11.84 |
| 20 |  | 11.84 |
| 2 |  | 15.30 |
| 2 |  | 15.10 |
| 0.2 |  | 18.13 |
| 0.2 |  | 18.28 |
| 0.02 | 18.39 | 21.28 |
| 0.02 | 18.27 | 21.24 |
| 0.002 | 19.98 |  |
| 0.002 | 19.91 |  |
| 0.00020 | 22.45 |  |
| 0.00020 | 22.37 |  |
| 0.00002 | 25.64 |  |
| 0.00002 | 25.59 |  |

Table SA1: Ct values for spiked cel-miR-39 in replicate qRT-PCR experiments in HUVEC and HPMEC using Applied Biosystem assay ID 000200. Concentrations were derived from Ct values by log10(nM) =-2.172449-(0.4466193)Ct.

RNA-seq
Our original focus was on EC from pulmonary and systemic micro-vasculatures. Given the genomic differences between individuals,[4,5] our original intention had been to obtain HDMEC and HPMEC from the same donor before same- and wider-donor qRT-PCR validations. However, in the relevant period, it was not feasible to source HDMEC and HPMEC from the same donor so these microvascular ECs were derived from separate donors, and both were treated in replicate wells for 1h with fresh media, CHX or ferric citrate. Both HDMEC and HPMEC were also treated for 6h with fresh media +/- ferric citrate or fresh media supplemented with CHX for the last 3h. As noted in the main text, 3h CHX resulted in morphological changes so those ECs were not processed for RNASeq.

Where bulk as opposed to single cell studies are performed, maximising the proportion of viable, healthy EC is important. We considered the trade-off in strategies between obtaining larger EC numbers by further passages or transformation (as [6,7]), versus examining untransformed EC at earlier passages when fewer cells have started to enter senescence. We selected an untransformed, earlier passage strategy that enabled triplicate wells for treatments- to use for RNA-seq, qRT-PCR validations (including cel-miR-39 spiking described above), and protein extractions. This was similar to our later studies using patient-derived BOECs when defrosted low passage BOECs were split for same-passage RNASeq, pulse chase and bulk flow cytometry without intervening freezing,[2], or purely used for RNA-seq following multiple treatments with variation between replicate libraries used to define the least variable for Bonferroni corrections.[2] For the current study, BOEC RNA yields were sufficiently high for Genewiz to generate two libraries per sample, targeting long RNAs (mRNA, lncRNA including pre-miRs) as described,[2] and small RNAs (miRNA, siRNA, piRNA) following size fractionation with adapter ligation to 5’ phosphates.

## Selection of cycloheximide (CHX)-enriched exons

Alignments to 4,246 exons were observed only in the CHX-treated cells (Figure 3A). Novel, alternative exons were selected if there were more than 20 reads, reducing the list to 24 exons (Table S4). Following this, exons of genes with poorly characterised functions and exon extensions were excluded. Finally, exons that introduced a premature termination codon (PTC) were positively selected by excluding exons that neither had a stop codon in each reading frame, nor induced a frameshift leading to the
formation of a PTC in the downstream exon.

This allowed the construction of a final prioritized list that comprised 5 exons from *SLC11A2, SLC2A5, SLMO1, PARD6G* and *AFAP1L.* Oligonucleotide primers were designed for the alternative exons inducing PTCs in *SLC11A, SLC2A5, SLMO1*, *PARD6G* and *AFAP1L1*. The qRT-PCR products were size-separated using low melt agarose gel electrophoresis to confirm nucleic acid sizes (Figure 4B). The ∆∆Ct method[8] was used to determine fold changes. Alignments to the CHX-enriched exon, and nearest adjacent exon for the best validated exons by qRT-PCR were examined in untreated further ECs using the Integrated Genome Browser (IGB).[9]

## Peripheral blood mononuclear cell (PBMC) expression

To test wider potential significance, *SLC11A2* and *pre-let-7b* (‘let-7b) were also examined in rRNA-depleted RNA-seq data from peripheral blood mononuclear cells (PBMCs) obtained from 6 donors.[4] Freshly derived PBMCs were resuspended in endogenous plasma, and distributed to separate experimental treatment tubes each containing ~5mL of cell/plasma suspension.[4] For the current study, we examined data from PBMCs treated with CHX to final concentration 100μg/mL, and PBMCs treated with an equivalent volume (<100μL) of Dulbecco´s Modified Eagle Medium (DMEM) supplemented with 10% fetal calf serum (FCS). After CHX or sham treatments, PBMCs were cultured at 37^o^C for 1h before centrifugation, resuspension of the cell pellet in TRI reagent (Sigma-Aldrich, UK), RNA extraction, ribosomal (r)RNA depletion, and generation of libraries for Illumina HiSeq sequencing using paired-end 150bp reads (Genewiz, Leipzig, Germany).[4] Genewiz also performed differential exon expression using DEXSeq[10] on exon regions and junctions of all genes, with *SMAD4* examples shown by Xiao et al [4]. Expression of *let-7b* and *SLC11A2* are displayed in Figure S2 and Figure S4 respectively.

# TABLES

## Table S1. Representative pathway terms identified through clustering of differentially-expressed genes after CHX or ferric citrate.

|  | **Source** | **Term** |  | **Genes** | **p-value** |  | **Benjamini** |  |  |  |  |
| --- | --- | --- | --- | --- | --- | --- | --- | --- | --- | --- | --- |
|  | **Ai) Cycloheximide (CHX) 1h, N=100 most differentially expressed genes** | | |  | |  | |  | | | |
|  | KEGG_PATHWAY hsa05224 | | Breast cancer | *DLL4, DVL2, FOS, POLK, AXIN2, LRP6* | | 0.00048 | | 0.21 | | | |
|  | KEGG_PATHWAY hsa05226 | | Gastric cancer | *DVL2, POLK, AXIN2, LRP6* | | 0.0029 | | 0.99 | | | |
|  | UP_SEQ_FEATURE DOMAIN | | EGF-like 3 * | *DLL4, LTBP4, LTBP3, LRP6* | | 0.0048 | | 1.0 | | | |
|  | UP_KW_PTM KW-0832 | | Ubl conjugation | *PHF3, CD274, BLM, PPP1R10, NSMCE1, DTNBP1, JMJD1C, RSRC2, ARID4B, TTF2, FOS, AXIN2, MYSM1, LRP6, ZBTB2, SLC7A5, MYBBP1A, ZC3H12A, UBC, EPC1, RP9, IRF6, PELP1* | | 0.0065 | | 0.052 | | | |
|  | UP_SEQ_FEATURE CROSSLNK | | Glycyl lysine isopeptide (Lys-Gly) (interchain with G-Cter in SUMO2) | *PHF3, BLM, MYBBP1A, PPP1R10, EPC1, JMJD1C, RP9, ARID4B, FOS, TTF2, MYSM1, ZBTB2* | | 0.012 | | 1.0 | | | |
|  | UP_KW_PTM KW-1017 | | Isopeptide bond | *PHF3, BLM, PPP1R10, JMJD1C, RSRC2, ARID4B, TTF2, FOS, MYSM1, LRP6, ZBTB2, SLC7A5, MYBBP1A, UBC, EPC1, RP9, PELP1* | | 0.015 | | 0.08 | | | |
|  | GOTERM_MF_DIRECT GO:0046872 | | Metal ion binding | *PHF3, PPP1R10, NSMCE1, HBB, JMJD1C, ZNF75D, DEF8, PTGS2, KALRN, MYSM1, ZBTB2, PPM1A, DGKQ, ZC3H12A, PDE4B, RP9, POLK, ATP7A, CBLL1, MAP3K4* | | 0.013 | | 1.0 | | | |
|  | GOTERM_BP_DIRECT GO:0045944 | | Positive regulation of transcription from RNA polymerase II promoter | *MYBBP1A, ZC3H12A, DVL2, FOSB, EPC1, ARID4B, IRF6, FOS, MYSM1, IER2, PELP1, LRP6* | | 0.012 | | 0.90 | | | |
|  | KEGG_PATHWAY hsa05217 | | Basal cell carcinoma | *DVL2, POLK, AXIN2* | | 0.032 | | 0.99 | | | |
|  | KEGG_PATHWAY hsa05225 | | Hepatocellular carcinoma | *DVL2, POLK, AXIN2, LRP6* | | 0.040 | | 0.99 | | | |
|  | GOTERM_MF_DIRECT GO:0003677 | | DNA binding | *BLM, MYBBP1A, PPP1R10, ZC3H12A, FOSB, ARID4B, IRF6, TTF2, ZNF75D, MYSM1, IER2* | | 0.047 | | 1.0 | | | |
| **Aii) CHX 1h and other NMD Datasets, N=37 shared differentially expressed genes** | | | |  |  | | | |  | |  |
| UP_SEQ_FEATURE DOMAIN |  |  | Helicase C-terminal | *DHX57, CHD6, INO80, SMARCA1* |  | 7.640E-4 |  | 0.078 |  |  |  |
| INTERPRO, IPR014001 |  |  | Helicase, superfamily 1/2, ATP-binding domain 4 | *DHX57, CHD6, INO80, SMARCA1* |  | 9.959E-4 |  | 0.062 |  |  |  |
| SMART, SM00490 |  |  | Helicase superfamily C-terminal domain | *DHX57, CHD6, INO80, SMARCA1* |  | 0.00137 |  | 0.023 |  |  |  |
| GOTERM_MF_DIRECT GO:0008094 |  |  | DNA-dependent ATPase activity | *CHD6, INO80, SMARCA1* |  | 0.0020 |  | 0.21 |  |  |  |
|  | GOTERM_MF_DIRECT GO:0005524 | | ATP binding 9 | *DHX57, ADK, CHD6, ABCA7, INO80, SMARCA1,  ABCC10, CLK4, MAP3K14* | | 0.0034 | | 0.21 | | | |
|  | INTERPRO, IPR027417 | | P-loop containing nucleoside triphosphate hydrolase | *DHX57, CHD6, ABCA7, INO80, SMARCA1,  ABCC10, RHOBTB1* | | 0.0051 | | 0.12 | | | |
|  | UP _KW_LIGAND KW-0067 | | ATP-binding | *DHX57, ADK, CHD6, ABCA7, INO80, SMARCA1, ABCC10, CLK4, MAP3K14* | | 0.0058 | | 0.03 | | | |

| **…/ Source** | **Term** | | **Genes** | | **p-value** | | **Benjamini** | |  |
| --- | --- | --- | --- | --- | --- | --- | --- | --- | --- |
| **Bi) Iron treatment 1h, N=100 most differentially expressed genes** | | |  | |  | |  | |  |
| GOTERM_MF_DIRECT GO:0051015 | | Actin filament binding | *CYFIP1, MACF1, MYH9, MYO5A, NEB, UTRN, SYNE1* | | | 0.00024 | | 0.032 | |
| GOTERM_MF_DIRECT GO:0003779 | | Actin binding | *MACF1, DST, MYH9, MYO5A, NEB, UTRN, SYNE1* | | | 0.0018 | | 0.071 | |
| GOTERM_CC_DIRECT GO:0005856 | | Cytoskeleton | *MACF1, KRT18, DST, USP9X, AKAP9, UTRN, LRPPRC, SYNE1* | | | 0.0047 | | 0.11 | |
| GOTERM_CC_DIRECT GO:0015629 | | Actin cytoskeleton | *MACF1, DST, MYH9, MYO5A, NEB* | | | 0.018 | | 0.27 | |
| GOTERM_MF_DIRECT GO:0003774 | | Motor activity | *CENPE, MYH9, MYO5A* | | | 0.023 | | 0.56 | |
| GOTERM_MF_DIRECT GO:0016887 | | ATPase activity | *DYNC1H1, PSMC4, YTHDC2, RAD54L, ATP7A, SMC4* | | | 0.025 | | 0.56 | |
| GOTERM_CC_DIRECT GO:0042995 | | Cell projection | *MACF1, RRM1, DST, UTRN* | | | 0.039 | | 0.37 | |
| **Bii) Iron treatment 6h, N=100 most differentially expressed genes** | | |  | | |  | |  | |
| UP_SEQ_FEATURE DOMAIN | | Helicase ATP-binding * | *DHX30, BLM, RTEL1, DDX49, DHX8, DDX55, DHX57, DHX38* | | | 5.2 x10^-7^ | | 0.00034 | |
| INTERPRO IPR002464 | | DNA/RNA helicase, ATP-dependent, DEAH-box type, conserved site * | *DHX30, BLM, DHX8, DHX57, DHX38* | | | 2.5 x10^-6^ | | 0.00066 | |
| GOTERM_MF_DIRECT GO:0003676 | | Nucleic acid binding | *DDB1, DHX30, RTEL1, DDX49, DHX8, RAVER1, DDX55, TDG, DHX57, DHX38* | | | 0.00001 | | 0.002 | |
| GOTERM_MF_DIRECT GO:0003724 | | RNA helicase activity | *DHX30, DDX49, DHX8, DDX55, DHX57, DHX38* | | | 0.000017 | | 0.002 | |
| UP_SEQ_FEATURE CROSSLNK: | | Glycyl lysine isopeptide (Lys-Gly) (interchain with G-Cter in SUMO2) | *BLM, DHX8, YLPM1, VRK1, UACA, FTSJ3, DDB1, KRT18, MYBBP1A, ZMIZ2, TDG, POLR3D, DHX38, EPC1, ZNF205, DMAP1, DUS3L* | | | 0.00044 | | 0.0065 | |
| GOTERM_MF_DIRECT GO:0016887 | | ATPase activity | *DYNC1H1, DHX30, BLM, RTEL1, DDX49, DHX8, DDX55, DHX57, DHX38* | | | 0.00060 | | 0.048 | |
| GOTERM_CC_DIRECT GO:0005654 | | Nucleoplasm | *BLM, RTEL1, DDX49, DHX8, GPS1, PTTG1IP, UACA, CNDP2, TDG, ZMIZ2, EP300, DHX38, EPC1, DMAP1, DST, WDR18, DDX55, YLPM1, VRK1, ARID1A, RABGGTA, PSMB8, FANCG, FTSJ3, DDB1, MYBBP1A, POLR3D, PKN2* | | | 0.0022 | | 0.14 | |
| GOTERM_MF_DIRECT GO:0003684 | | Damaged DNA binding | | *DDB1, TDG, EP300, FANCG* | | 0.0029 | | 0.10 | |
| UP_KW_PTM KW-1017 | | Isopeptide bond | | *BLM, DST, DHX8, YLPM1, VRK1, UACA, FTSJ3, DDB1, KRT18, MYBBP1A, ZMIZ2, TDG, POLR3D, EP300, DHX38, EPC1, ZNF205, DMAP1, DUS3L* | | 0.0054 | | 0.037 | |
| GOTERM_BP_DIRECT GO:0006974 | | Cellular response to DNA damage stimulus | | *DDB1, BLM, VRK1, FANCG, FBXO31* | | 0.031 | | 1.0 | |
| GOTERM_CC_DIRECT GO:0000781 | | Chromosome, telomeric region | | *DDB1, BLM, RTEL1, TEP1* | | 0.031 | | 0.82 | |
| GOTERM_BP_DIRECT GO:0006281 | | DNA repair | | *DDB1, BLM, RTEL1, DMAP1, FANCG* | | 0.032 | | 1.00 | |
| UP_KW_PTM KW-0832 | | Ubl conjugation | | *OFD1, BLM, DST, DHX8, YLPM1, VRK1, UACA, TBC1D3H, FTSJ3, DDB1, KRT18, MYBBP1A, ZMIZ2, TDG, POLR3D, EP300, DHX38, EPC1, ZNF205, DMAP1, DUS3L* | | 0.047 | | 0.21 | |

**Representative pathway terms identified through differentially-expressed genes.** Clustering was performed using the Database for Annotation, Visualization and Integrated Discovery [11] after **A)** cycloheximide or **B)** ferric citrate 10μmol/L. Table indicates source of gene term in cluster,[12-16] name of term, differentially expressed genes in the cluster, the crude p value and the Benjamini-adjusted p value. **A)** Cycloheximide 1h 100μg/mL: **i)** terms derived from top 100 differentially expressed genes in HDMEC after 1hr (data from top 419 genes in Figure S1), and **ii)** the 37 genes that were differentially expressed to p<0.15 in HDMEC and also differentially expressed post inhibition of nonsense mediated decay (NMD) by *SMG5*, *SMG6* or *SMG7* knockdown in mouse embryonic stem cells,[17,18] or after siRNA inhibition of *UPF1* in human HeLa cells.[7] Representative terms in the only significant cluster (enrichment score 1.85) are presented. **B)**  Representative terms and cluster genes after **i)** 1h and **ii)** 6h treatment with 10μmol/L ferric citrate. *Highly similar terms of lower significance from the same annotation methods are not shown.

## Table S2 (abridged): Details of let-7 Target genes in 6hr data sets identified by TargetScan

| **A) Genes with >1 let-7 binding site** | | **Conserved sites** | | | | **Poorly  conserved sites** | \| **Example miR** \| \| --- \| \|  \| | **Total Context Score** | **Aggregate PCT** |
| --- | --- | --- | --- | --- | --- | --- | --- | --- | --- | --- | --- |
| **Gene** | **Gene Name** | **total** | **8mer** | **7mer-m8** | **7mer-1A** |  |  |  |  |
| *PUNC* | Putative neuronal cell adhesion molecule | 3 | 3 | 0 | 0 | 1 | hsa-let-7i | -0.72 | > 0.99 |
| *IGF2BP1* | Insulin-like growth factor 2 mRNA binding protein 1 | 5 | 3 | 1 | 1 | 0 | hsa-let-7d | -0.82 | > 0.99 |
| *SMC1A* | Structural maintenance of chromosomes 1A | 2 | 2 | 0 | 0 | 1 | hsa-let-7a | -0.49 | 0.97 |
| *BACH1* | BTB and CNC homology 1, basic leucine zipper transcription factor 1 | 2 | 2 | 0 | 0 | 0 | hsa-let-7c | -0.47 | > 0.99 |
| *LIN28B* | Lin-28 homolog B (C. elegans) | 4 | 2 | 2 | 0 | 1 | hsa-let-7d | -1.12 | > 0.99 |
| **B) Genes with 1 let-7 binding site**  See separate pdf for full list at end of Data Supplement as Table S2 (full). | |  |  |  |  |  |  |  |  |

TargetScan 5.2 [19] identified from 17,821 human mRNAs, 819 conserved let-7 targets with a total of 905 conserved and 125 poorly conserved sites, where the basic seed-based algorithm is from Lewis et al,[20] context scores from Grimson et al,[21] and the use of preferential conservation (branch length and PCT) from Friedman et al.[22] Site conservation was defined by conserved branch length, which was for 8mer ≥ 0.8; 7mer-m8≥1.3, and 7mer-1A ≥1.6. Of these 819 mRNAs, 570 (69.6%) were present in the 10,819 mRNAs identified in media and 6h ferric citrate-treated HPMECs, where RNA-seq fold changes after 6h treatment are displayed in Figure 2C, compared to the other 10,279 mRNAs detected in the 0h and 6h ferric citrate-treated HPMECs. Specific metrics for these 570 mRNAs are listed in the above abridged table, and in full at the end of the Data Supplement.

## Table S3: Small RNA sequencing for mature let7-b miRNAs in endothelial cells

| Mature 5’ (let -7b-5p) | | | | Mature 3’ (let-7b-3p) | | | | Other | | Total |
| --- | --- | --- | --- | --- | --- | --- | --- | --- | --- | --- |
| Mature | Unique mature | Exact mature | Unique exact mature* | Mature | Unique mature | Exact mature | Unique exact mature* | Exact | Other |  |
| 8529 | 8486 | 6397 | 6397 | 375 | 375 | 1 | 1 | 66 | 84 | 8988 |
| 12674 | 12592 | 9544 | 9544 | 365 | 365 | 0 | 0 | 131 | 165 | 13204 |
| 10308 | 10237 | 7649 | 7649 | 260 | 260 | 0 | 0 | 55 | 96 | 10664 |
| 11692 | 11623 | 8645 | 8645 | 372 | 372 | 0 | 0 | 111 | 140 | 12204 |

It is known that for any given miRNA, there can be a strong strand bias, due to degradation of the passenger strand that is not loaded into argonaute (AGO) proteins.[23,24]

Here, raw expression values for total, exact, unique and unique exact matches to let-7b-5p and let-7b-3p small RNA sequencing of 4 healthy donor BOEC cultures are provided, as generated by Genewiz (Leipzig, Germany) who size-fractionated RNAs from the same 4 normal volunteer BOEC cultures as also used for rRNA-depletion/standard RNA-seq. Following size fractionaton, adapters were ligated to the 5’ phosphate, prior to library generation.

* Data plotted in Figure 3 (for the unique, exact mature expression values for the 5p and 3p mature miRNAs).

## Table S4: Novel alternate exons in CHX-treated HDMEC

| **Gene** | **ExonID** | **GeneID** | **Exon** | **Length** | **Chr** | **Strand** | **Start** | **End** | **Sequence** |
| --- | --- | --- | --- | --- | --- | --- | --- | --- | --- |
| *SLC11A2* | 199534 | 12155 | 3B | 25 | chr12 | - | 49685703 | 49685727 | AATAAGAGGCTGATGGAACCTGCAG |
| *SLC2A5* | 26066 | 1636 | U8 | 56 | chr1 | - | 9054950 | 9055005 | GCTGGAGTGCAGTGGCATGATCGAATTCCTGGGCTCAAGCGATCCTCTTGCCTCAG |
| *PARDG6* | 262884 | 15890 | 2A | 55 | chr18 | - | 76060838 | 76060892 | GTGGTGAACACTGACCACCTCAAGGAACCAAGCAGCCAGTGGTGACAGTTGCAAG |
| *SLMO1* | 263721 | 15939 | 1B | 56 | chr18 | + | 12409891 | 12409946 | GCACTCCGGCCTGGGTGACAGAGCAAGACTCCGTCTCAAACAAAAAAGTCATTCAG |
| *AFAP1L1* | 84006 | 4974 | 5A | 50 | chr5 | + | 148664027 | 148664076 | CTGATGGACCTTGGGCTGATTGCACCTTTTGGCTATGGTGCTTGGATCAG |
| *Not prioritised* | |  |  |  |  |  |  |  |  |
| *NUCB1* | 274872 | 16698 | 8A' | 33 | chr19 | + | 54114147 | 54114179 | ATGGAGGAGGAGCGACTGCGCATGCGGGAGCAT |
| *TIA1* | 51127 | 3043 | 4A | 33 | chr2 | - | 70309695 | 70309727 | GTAGTACCGTTGTCAGCACACAGCGTTCACAAG |
| *TNXB* | 116531 | 7005 | U3 | 38 | chr6 | - | 32203238 | 32203275 | TTTGACGGCAGCTCCCTGGACGTGGGGATGGATGTCAG |
| *SFI1* | 299986 | 18375 | U1 | 42 | chr22 | + | 30152727 | 30152768 | CCTATTGGAAAAGATCTGGGACTATCTGAAACTAGTGAGAAT |
| *OVOL2* | 287577 | 17608 | W2 | 59 | chr20 | - | 17885623 | 17885681 | CTGAGATCTGTATCTGTGGACCTGAATGTTGATCCCTCGCTTCAGATTGACATACCTGA |
| *RAD51L1* | 214776 | 13080 | 10A | 45 | chr14 | + | 68005025 | 68005069 | ATCCTATGGCATGAAGTGGGCAATGATTCTGACACCAGTGTGGAG |
| *MX1* | 293019 | 17978 | U4 | 45 | chr21 | + | 41715126 | 41715170 | GACGGGCAGGAGACAGATGCCTTCCTCTTGTCTCAACTGCAAGAG |
| *MPV17L* | 235978 | 14243 | W1 | 46 | chr16 | + | 15516663 | 15516708 | ATCAGCTGGACATCATCTCCATGGCGGAGACAACCATGATGCCAGA |
| *WASF1* | 118141 | 7091 | 2A | 57 | chr6 | - | 110605643 | 110605699 | CTTGTTGCATTTACCCTTTTGATAAAAGAGAATCACATAAGCATTGCAGAGGCAGTT |
| *ALDH3B2* | 168876 | 10159 | U1 | 49 | chr11 | - | 67205223 | 67205271 | CTGCCACCATGTGAAGAAGGATGTGGTTGCTTCCCCTTCCACCATAACT |
| *SIRT3* | 182299 | 11146 | 2b' | 49 | chr11 | - | 223438 | 223486 | TGTTGTTGGAAGTGGAGGCAGCAGTGACAAGGGGAAGCTTTCCCTGCAG |
| *MTRF1* | 205777 | 12538 | 1B | 71 | chr13 | - | 40734547 | 40734617 | GAAAGTATTCCATGCTACATGTGCAAGAAACTTGGAAAACACTGAAAAGTAGAAAAA ATAGCAAGCAAAAG |
| *CHID1* | 171768 | 10341 | 2A | 50 | chr11 | - | 893704 | 893753 | GCTGGAGCGCAATGGCGCGATCTTGGCTCACCGCAACCTCTGCCACCCAG |
| *TJAP1* | 116322 | 6990 | 2a' | 50 | chr6 | + | 43553803 | 43553852 | GCTTAGATCAGCCTTTCCACAGCTGTTAGCAGCATCTGCCCCAATTTCAG |
| *DNAJC4* | 173081 | 10415 | 4c' | 80 | chr11 | + | 63756478 | 63756557 | CTGCACCCAGACCGGGACCCTGGGAACCCAAGCCTGCACAGCCGCTTTGTGGAGCTGA GCGAGGCATACCGTGTGCTCAG |
| *BMP4* | 208853 | 12715 | 3a' | 53 | chr14 | - | 53488645 | 53488697 | AGACACCATGATTCCTGGTAACCGAATGCTGATGGTCGTTTTATTATGCCAAG |
| *TIAM2* | 116268 | 6988 | U6 | 95 | chr6 | + | 155376748 | 155376842 | ACACACACACACACACACACACACACACACACCTGAGATGGGGTAGATCATTGTATTT TTGTGTCTACCAGCAAGAAAAGGAAGGAAAAACTAAG |
| *MTP18* | 298212 | 18272 | 3b' | 69 | chr22 | + | 29153158 | 29153226 | GTGCCCAGCCCTGAAGCAGGCCGCAGCGCCAGGGTGACTGTGGCTGTGGTGGACACC TTTGTATGGCAG |
| *SEZ6* | 255662 | 15470 | 8a' | 55 | chr17 | - | 24310982 | 24311036 | CCTTCCAGCAGGGCCATTGCTATGAGCCCTTTGTCAAATACGGTAACTTCAGCAG |

The 24 exons identified only in CHX-treated HDMEC with >20 reads**.** Chr, chromosome. Start and end coordinates as aligned to GRCh18. Primers sequences available on request.

## Table S5. Iron Prescription Data in England, financial year 2022/2023

| **Chemical Substance Name** | **Total Items** | **Elemental iron (mg)** |
| --- | --- | --- |
| Ferrous fumarate | 4,832,062 | 69-106 * |
| Ferrous sulfate | 3,240,145 | 65-105 * |
| Ferrous gluconate | 276,501 | 35 * |
| Ferric maltol | 12,321 | 30 * |
| Other oral iron preparations | 15,102 | vary |
| Iron and folic acid | 14,780 | vary |
| Sodium feredetate | 207,556 | liquid |
| Iron dextran | 400 | iv solution |
| Iron sucrose | 27 | iv solution |
| Iron carboxymaltose | 12 | Iv solution |

The first 2 columns are extracted directly from the NHS Business Services Authority’s 2022/2023 Statistical Summary Tables.[25] The third column was derived from per tablet content in compounds on the British National Formulary (BNF).[26] *BNF caution to “stop at at oral doses greater than 200 mg elemental iron daily (no evidence of enhanced iron absorption above these doses)”.

# FIGURES

# Figure S1: Comparisons of media and cycloheximide (CHX)-treated HDMEC


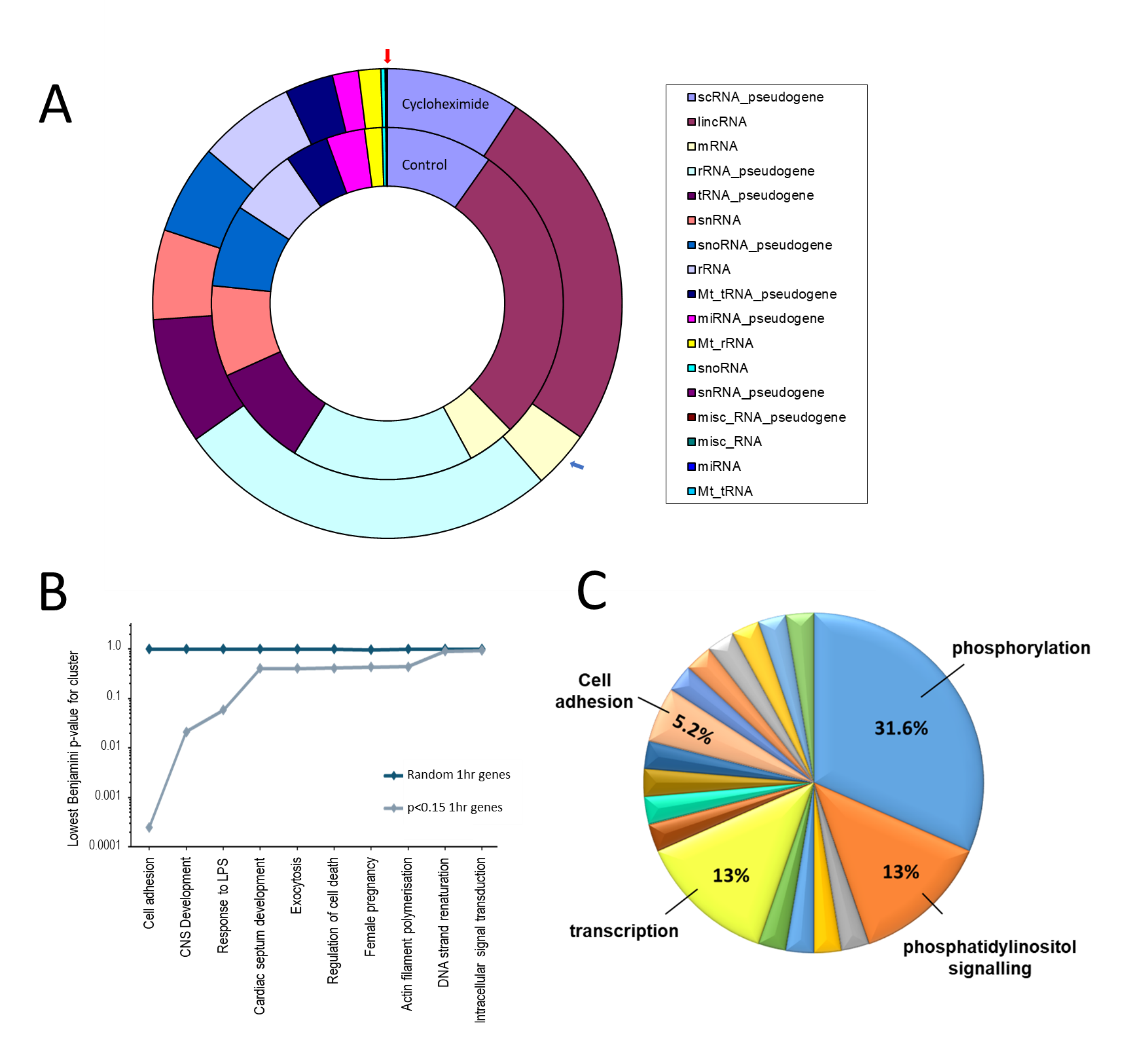


**A)** Alignment to different RNA types in HDMEC treated with 100μg/mL CHX or media for 1h. The outer ring represent library created for CHX-treated HDMEC. Red arrow indicates alignments to miRNAs.  **B)** From alignments to 15,756 genes, the 419 genes with the most differential alignments after 1h treatment with CHX or fresh media (where differential expression was determined at p<0.15) were clustered using DAVID v6.[11] To control for bias, clustering was also carried out on a random list of 419 genes generated from the CHX RNA-seq dataset. For each cluster, the most significant processes with the smallest Bonferroni p-values were selected, and p-values for the same processes in the random set of genes displayed.
**C)** Following identification of pre-miRNAs that demonstrated alignments at least 20% higher in CHX-treated HDMEC or media-treated HDMEC, target genes of pre-miRNAs were identified through TargetScan.[19] Target genes were categorised depending on the number of miRNA binding sites, and a maximum of 3000 targets were used for each of the miRNAs for Gene Ontology Clustering to find cellular processes with which they were associated.

##

## Figure S2: let-7b-5p expression in HDMECs, plasma and peripheral blood mononuclear cells

**
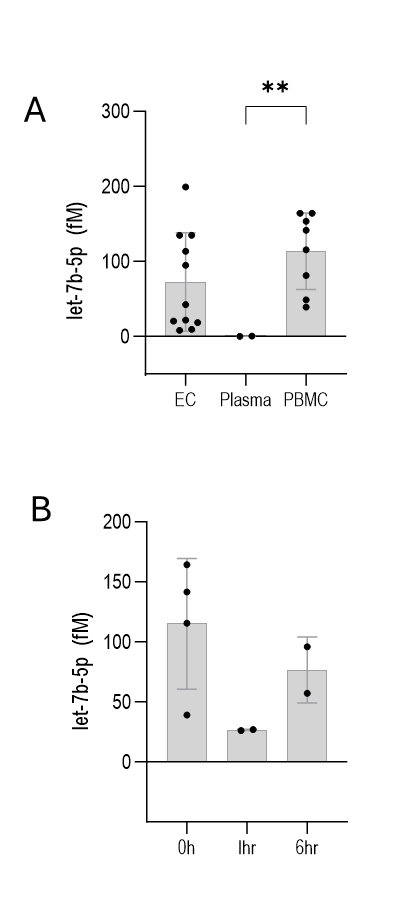
**

**A)** Expression of let-7b-5p as determined by qRT-PCR of RNA from untreated HDMECs, plasma and peripheral blood mononuclear cells (PBMCs) using Applied Biosystems miRNA assay 002619. Absolute concentrations were obtained by cel-miR-39 spikes as described in Supplementary Methods, quantified by using Applied Biosystem assay ID 000200. Note varying levelsof let-7b-5p in different donors’ PBMCs and ECs. Ct values were <26.3 in EC, <24.5 in PBMCs, and expression in these cells was always higher than in plasma (**, p<0.01 by Dunn’s test post Kruskal Wallis).

**B)** Expression of let-7b-5p in sham-treated PBMCs (0h) and PBMCs treated for 1h or 6h with 10μmol/L ferric citrate using Applied Biosystems miRNA assay 002619 after cel-miR-39 spikes and quantification using Applied Biosystem assay ID 000200. Note the trends paralleled those observed in HDMECs, HPMECs, HUVEC and BOECs shown in main manuscript Figure 2.

## Figure S3: Comparison of expression patterns of CHX-enriched exons in endothelial cells (BOECs)

**
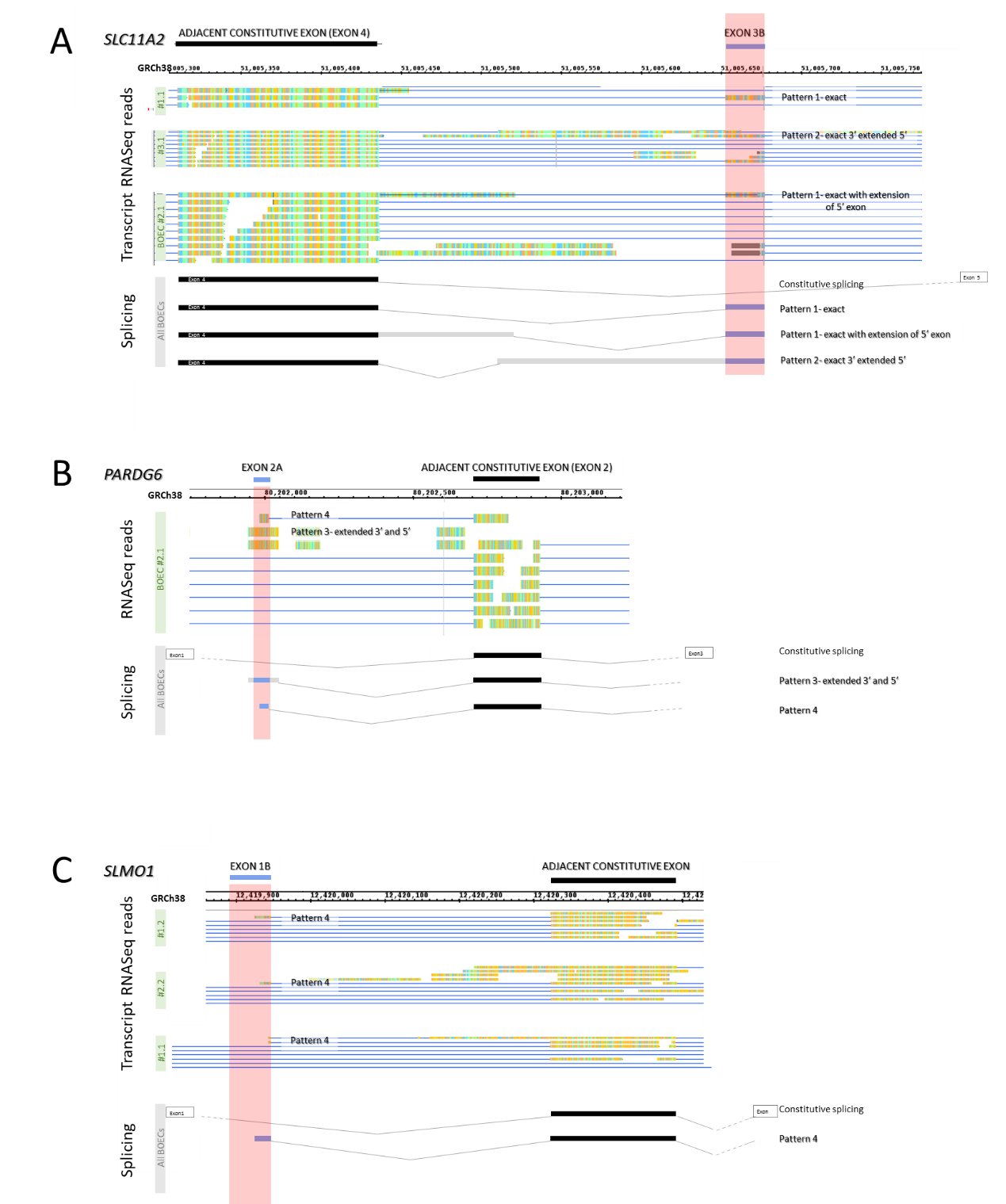
**

IGB 9.1.8 [9] views of GRCh38/hg38 [27] are accompanied by annotations of splicing patterns. For each gene, the position of the CHX-enriched exons are highlighted in red across separate BOEC sets of alignments including patient datasets to expand read numbers for**:**

**A)** Chr12:51,005,295-51,005,797 spanning the CHX-enriched *SLC11A2* exon 3B and adjacent constitutive exon 4 (reads are now annotated by pattern type, where precise splicing is listed as pattern 1).

**B)** Chr18:80,200,939-80,203,651 spanning CHX-enriched *PARD6G* exon 2A to adjacent constitutive exon 2.

**C)** Chr18:12,419,463-12,420,541 spanning the CHX-enriched *SLMO1*exon 1B and adjacent constitutive exon.

Note exon-exon boundary read through in **B)** and **C)**, and CHX-enriched exons absent from respective RefSeq transcripts denoted by constitutive splicing patterns. The other alignment patterns observed in the BOECs in addition to the precise splicing (pattern 1), were 5’ extension (pattern 2); both 5’ and 3’ extension (Pattern 3), and use as an alternate first exon (Pattern 4, Figure 4). *SLC11A2* exon 3B displayed predominantly Patterns 1 and 2, *PARD6G* exon 2A predominantly Pattern 3, and *SLM01* exon 1B predominantly Pattern 4.

## Figure S4: *SLC11A2* expression in human peripheral blood mononuclear cells (PBMCs) compared to endothelial cells

##
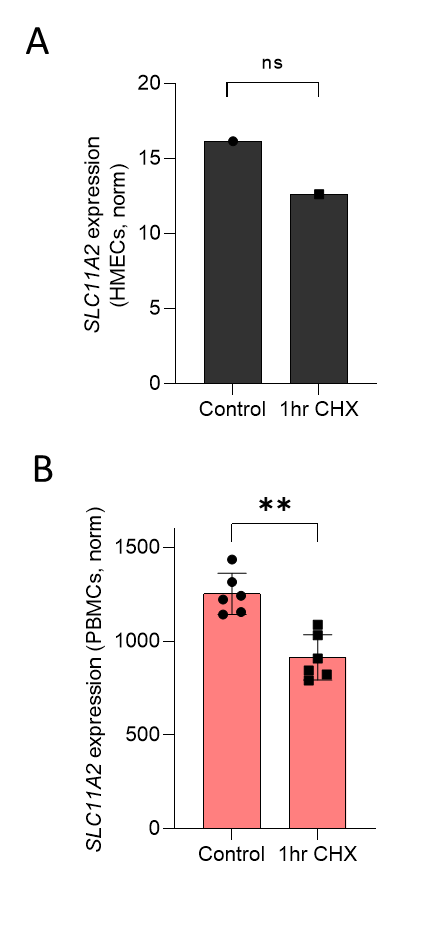


**A)** Endothelial expression of *SLC11A2*: Mean alignments in the original endothelial experiment in HDMEC, quantified across 18 exons and junctions in HDMEC treated with fresh media for 1h, and 20 exons and junctions in HDMEC after 1h treatment with 100μg/mL CHX. The apparent trend for fewer alignments post CHX did not meet significance.  **B)** PBMC: Normalised mean reads for *SLC11A2* expression in PBMCs resuspended in endogenous plasma after 1h incubation with and without 100μg/mL CHX. Note the similar decrement post CHX, and that in this cell type, using mean data from replicates in 6 donors (including 3 patients with HHT,[4] the difference was significant (p=0.0022 by Mann Whitney).

## **SUPPLEMENTARY REFERENCES:**

1 Shovlin CL, Angus G, Manning RA, Okoli GN, Govani FS, Elderfield K, et al. Endothelial cell processing and alternatively spliced transcripts of factor VIII: potential implications for coagulation cascades and pulmonary hypertension. *PLoS One* 2010;5(2): e9154.

2 Bernabeu-Herrero ME, Patel D, Bielowka AM, Zhu J, Jain K, Mackay IS, et al. Mutations causing premature termination codons discriminate and generate cellular and clinical variability in HHT. *Blood* 2024 May 30;143(22):2314-2331.

3 Kartikasari AE, Georgiou NA, Visseren FL, van Kats-Renaud H, van Asbeck BS, Marx JJ. Endothelial activation and induction of monocyte adhesion by non transferrin-bound iron present in human sera. *FASEB J* 2006;20: 353–5.

4 Xiao S, Kai Z, Murphy D, Li D, Patel D, Bielowka AM, et al. Functional filter for whole-genome sequencing data identifies HHT and stress-associated non-coding SMAD4 polyadenylation site variants >5 kb from coding DNA. *Am J Hum Genet* 2023 Nov 2;110(11):1903-1918.

5 Shovlin CL, Aldred MA. When “loss-of-function” means proteostasis burden: Thinking again about coding DNA variants (mutations). *Am J Hum Genet* 2024. in press.

6 Shovlin CL, Hughes JM, Scott J, Seidman CE, Seidman JG. Characterization of endoglin and identification of novel mutations in hereditary hemorrhagic telangiectasia. *Am J Hum Genet* 1997 Jul;61(1):68-79.

7 Mendell J., Sharifi NA, Meyers JL., Martinez-Murillo F, Dietz HC. *Nat Genet* 2004;36(10):1073-8.

8 Winer J, Jung CK, Shackel I, Williams PM. Development and validation of real-time quantitative reverse transcriptase-polymerase chain reaction for monitoring gene expression in cardiac myocytes in vitro. *Anal Biochem* 1999 May 15;270(1):41-9.

9 Freese NH, Norris DC, Loraine AE. Integrated genome browser: visual analytics platform for genomics. *Bioinformatics* 2016, 32(14), 2089-95.

10 Anders S, Reyes A, Huber W. Detecting differential usage of exons from RNA-seq data. *Genome Res* 2012; 22(10):2008-17.

11 Dennis G Jr, Sherman BT, Hosack DA, Yang J, Gao W, Lane HC, Lempicki RA. DAVID: Database for Annotation, Visualization, and Integrated Discovery. *Genome Biol* 2003;4(5):P3.

12 Kanehisa, M., Goto, S. KEGG: Kyoto Encyclopedia of Genes and Genomes. *Nucleic Acids Res* 2000,28,27-30.

13 UniProt Consortium. UniProt: the Universal Protein Knowledgebase in 2023. Nucleic Acids Res. 2023;51(D1):D523-D531.

14 Gene Ontology Consortium; Aleksander SA, Balhoff J, Carbon S, Cherry JM, Drabkin HJ,. The Gene Ontology knowledgebase in 2023. *Genetics* 2023;224(1):iyad031.

15 Letunic I, Khedkar S, Bork P. SMART: recent updates, new developments and status in 2020. *Nucleic Acids Res* 2021 Jan 8;49(D1):D458-D460.

16 Paysan-Lafosse T, Blum M, Chuguransky S, Grego T, Pinto BL, Salazar GA, et al. InterPro in 2022. *Nucl Acid Res* 2023;51(D1):D418-D427.

17 Huth M, Santini L, Galimberti E, Ramesmayer J, Titz-Teixeira F, Sehlke R, et al. *Genes Dev* 2022;36:348-67.

18 Lackner A, Sehlke R, Garmhausen M, Giuseppe Stirparo G, Huth M, Titz-Teixeira F et al. Cooperative genetic networks drive embryonic stem cell transition from naïve to formative pluripotency. *EMBO J* 2021;40(8):e105776.

19 McGeary SE, Lin KS, Shi CY, Pham TM, Bisaria N, Kelley GM, Bartel DP. The biochemical basis of microRNA targeting efficacy. *Science* 2019;366:eaav1741

20 Lewis BP, Burge CB, Bartel DP. Conserved seed pairing, often flanked by adenosines, indicates that thousands of human genes are microRNA targets. *Cell* 2005;120:15-20.

21 Grimson A, Farh KK, Johnston WK, Garrett-Engele P, Lim LP, Bartel DP. MicroRNA targeting specificity in mammals: determinants beyond seed pairing. *Molecular Cell* 2007; 27:91-105.

22 Friedman RC, Farh KK, Burge CB, Bartel DP. Most Mammalian mRNAs Are Conserved Targets of MicroRNAs*. Genome Res* 2009;19:92-105.

23 Meijer HA, Smith EM, Bushell M. Regulation of miRNA strand selection: follow the leader? *Biochem Soc Trans* 2014;42:1135–40.

24 O'Brien J, Hayder H, Zayed Y, Peng C. Overview of MicroRNA Biogenesis, Mechanisms of Actions, and Circulation. *Front Endocrinol (Lausanne)* 2018;9:402

25 NHS Business Services Authority. *Prescription costs analysis for England* 2022/23. Available at https://www.nhsbsa.nhs.uk/statistical-collections/prescription-cost-analysis-england/prescription-cost-analysis-england-2022-23, accessed 01 December 2024.

26 National Institute for Health and Care Excellence (NICE) . *The British National Formulary*. Available at https://bnf.nice.org.uk/, accessed 01 December 2024.

27 National Center for Biotechnology Information, U.S. National Library of Medicine: Genome Reference Consortium Human Build 38, available at <https://www.ncbi.nlm.nih.gov/datasets/genome/GCF_000001405.40/>, accessed 1 Dec 2024.
