# Supplementary material for: Acute endothelial stresses identify microRNA let-7b-5p and non-coding SLC11A2 (NRAMP2/DMT1) exon as biomarkers that overlap with those detected in malignant and non-malignant diseases
Source: QJM. 2024 Dec 10;118(9):679–88. doi: 10.1093/qjmed/hcae235 (PMC12668437; doi:10.1093/qjmed/hcae235)
Supplement: hcae235_Supplementary_Data [file hcae235_supplementary_data.zip › hcae235_Supplementary_Data/TABLE S2 in full.pdf]

Gene	Gene name	Conserved sites				Poorly conserved sites				Representative miRNA	Total context score	Aggregate PCT
		total	8mer	7mer-m8	7mer-1A	total	8merPC	7mer-m8	7mer-1A			
PUNC	putative neuronal cell adhesion molecule	3	3	0	0	1	0	1	0	hsa-let-7i	-0.72	> 0.99
IGF2BP1	insulin-like growth factor 2 mRNA binding protein 1	5	3	1	1	0	0	0	0	hsa-let-7d	-0.82	> 0.99
SMC1A	structural maintenance of chromosomes 1A	2	2	0	0	1	1	0	0	hsa-let-7a	-0.49	0.97
BACH1	BTB and CNC homology 1, basic leucine zipper transcription factor 1	2	2	0	0	0	0	0	0	hsa-let-7c	-0.47	> 0.99
LIN28B	lin-28 homolog B (C. elegans)	4	2	2	0	1	0	0	1	hsa-let-7d	-1.12	> 0.99
ABCC5	ATP-binding cassette, sub-family C (CFTR/MRP), member 5	1	1	0	0	0	0	0	0	hsa-let-7g	-0.3	0.97
AEN	apoptosis enhancing nuclease	1	1	0	0	1	0	1	0	hsa-let-7g	-0.29	0.82
AHCTF1	AT hook containing transcription factor 1	1	1	0	0	0	0	0	0	hsa-let-7f	-0.46	0.54
AKT2	v-akt murine thymoma viral oncogene homolog 2	1	1	0	0	0	0	0	0	hsa-let-7i	-0.14	0.65
AMMECR1L	AMME chromosomal region gene 1-like	1	1	0	0	0	0	0	0	hsa-let-7d	-0.09	< 0.1
ANKFY1	ankyrin repeat and FYVE domain containing 1	1	1	0	0	0	0	0	0	hsa-let-7a	-0.08	0.94
ANKRD28	ankyrin repeat domain 28	1	1	0	0	0	0	0	0	hsa-let-7a	-0.23	0.71
ANKRD49	ankyrin repeat domain 49	1	1	0	0	0	0	0	0	hsa-let-7g	-0.26	0.69
APBB3	amyloid beta (A4) precursor protein-binding, family B, member 3	1	1	0	0	0	0	0	0	hsa-let-7a	-0.38	0.98
ARHGAP28	Rho GTPase activating protein 28	1	1	0	0	0	0	0	0	hsa-let-7a	-0.39	0.98
ARPP-19	cyclic AMP phosphoprotein, 19 kD	1	1	0	0	0	0	0	0	hsa-miR-98	-0.21	0.98
ATAD2B	ATPase family, AAA domain containing 2B	1	1	0	0	0	0	0	0	hsa-let-7d	-0.11	0.5
ATG4B	ATG4 autophagy related 4 homolog B (S. cerevisiae)	1	1	0	0	0	0	0	0	hsa-let-7a	-0.15	< 0.1
ATPAF1	ATP synthase mitochondrial F1 complex assembly factor 1	1	1	0	0	1	0	1	0	hsa-let-7d	-0.61	0.9
B3GNT1	UDP-GlcNAc:betaGal beta-1,3-N-acetylglucosaminyltransferase 1	1	1	0	0	0	0	0	0	hsa-let-7f	-0.29	0.73
BCAT1	branched chain aminotransferase 1, cytosolic	1	1	0	0	0	0	0	0	hsa-let-7g	-0.43	0.99
BCL2L1	BCL2-like 1	1	1	0	0	0	0	0	0	hsa-let-7b	-0.13	0.84
BRF2	BRF2, subunit of RNA polymerase III transcription initiation factor, BRF1-like	1	1	0	0	0	0	0	0	hsa-let-7d	-0.41	< 0.1
C15orf39	chromosome 15 open reading frame 39	1	1	0	0	0	0	0	0	hsa-let-7a	-0.26	0.85
C1orf91	chromosome 1 open reading frame 91	1	1	0	0	0	0	0	0	hsa-let-7a	-0.35	0.83
C22orf9	chromosome 22 open reading frame 9	1	1	0	0	1	0	0	1	hsa-let-7a	-0.06	> 0.99
C8orf58	chromosome 8 open reading frame 58	1	1	0	0	0	0	0	0	hsa-let-7d	-0.41	0.93
CANT1	calcium activated nucleotidase 1	1	1	0	0	0	0	0	0	hsa-let-7b	-0.17	< 0.1
CASP3	caspase 3, apoptosis-related cysteine peptidase	1	1	0	0	0	0	0	0	hsa-let-7b	-0.38	0.98
CCDC93	coiled-coil domain containing 93	1	1	0	0	0	0	0	0	hsa-let-7a	-0.17	0.52
CCNF	cyclin F	1	1	0	0	0	0	0	0	hsa-let-7d	-0.34	0.83
CCNJ	cyclin J	1	1	0	0	1	0	0	1	hsa-let-7g	-0.54	0.94
CDKN1A	cyclin-dependent kinase inhibitor 1A (p21, Cip1)	1	1	0	0	0	0	0	0	hsa-let-7i	-0.25	0.77
CDV3	CDV3 homolog (mouse)	1	1	0	0	0	0	0	0	hsa-let-7c	-0.36	0.98
CEP110	centrosomal protein 110kDa	1	1	0	0	0	0	0	0	hsa-let-7a	-0.4	0.96
CHIC1	cysteine-rich hydrophobic domain 1	1	1	0	0	1	0	0	1	hsa-miR-98	-0.09	0.85
CLDN12	claudin 12	1	1	0	0	0	0	0	0	hsa-let-7a	-0.39	0.95
COIL	coilin	1	1	0	0	0	0	0	0	hsa-let-7f	-0.5	0.92
COL1A2	collagen, type I, alpha 2	1	1	0	0	0	0	0	0	hsa-let-7g	-0.45	0.93
COL3A1	collagen, type III, alpha 1	1	1	0	0	0	0	0	0	hsa-let-7a	-0.42	0.93
COL4A6	collagen, type IV, alpha 6	1	1	0	0	0	0	0	0	hsa-let-7a	-0.32	0.91
COL5A2	collagen, type V, alpha 2	1	1	0	0	0	0	0	0	hsa-let-7d	-0.2	0.98
CPEB1	cytoplasmic polyadenylation element binding protein 1	1	1	0	0	0	0	0	0	hsa-let-7a	-0.42	0.88
DAGLA	diacylglycerol lipase, alpha	1	1	0	0	0	0	0	0	hsa-let-7d	-0.21	0.8
DCUN1D3	DCN1, defective in cullin neddylation 1, domain containing 3 (S. cerevisiae)	1	1	0	0	0	0	0	0	hsa-let-7d	-0.21	0.98
DDI2	DDI1, DNA-damage inducible 1, homolog 2 (S. cerevisiae)	1	1	0	0	0	0	0	0	hsa-let-7g	-0.36	0.86
DDX19A	DEAD (Asp-Glu-Ala-As) box polypeptide 19A	1	1	0	0	0	0	0	0	hsa-let-7g	-0.39	0.98
DDX19B	DEAD (Asp-Glu-Ala-As) box polypeptide 19B	1	1	0	0	0	0	0	0	hsa-let-7g	-0.39	0.9
DLGAP4	discs, large (Drosophila) homolog-associated protein 4	1	1	0	0	0	0	0	0	hsa-let-7d	-0.25	0.8
DMD	dystrophin (muscular dystrophy, Duchenne and Becker types)	1	1	0	0	0	0	0	0	hsa-let-7d	-0.35	0.98
DNA2	DNA replication helicase 2 homolog (yeast)	1	1	0	0	0	0	0	0	hsa-let-7f	-0.58	0.49
DOT1L	DOT1-like, histone H3 methyltransferase (S. cerevisiae)	1	1	0	0	0	0	0	0	hsa-let-7a	N/A	0.98
DPP3	dipeptidyl-peptidase 3	1	1	0	0	0	0	0	0	hsa-let-7a	-0.33	0.74
DVL3	dishevelled, dsh homolog 3 (Drosophila)	1	1	0	0	0	0	0	0	hsa-let-7f	-0.38	0.77
E2F6	E2F transcription factor 6	1	1	0	0	0	0	0	0	hsa-let-7c	-0.29	0.94
EDEM3	ER degradation enhancer, mannosidase alpha-like 3	1	1	0	0	0	0	0	0	hsa-let-7a	-0.27	0.98
EDN1	endothelin 1	1	1	0	0	0	0	0	0	hsa-miR-98	-0.35	0.82
EFHD2	EF-hand domain family, member D2	1	1	0	0	0	0	0	0	hsa-let-7f	-0.2	0.62
EGR3	early growth response 3	1	1	0	0	0	0	0	0	hsa-let-7a	-0.03	0.22
EIF2S2	eukaryotic translation initiation factor 2, subunit 2 beta, 38kDa	1	1	0	0	0	0	0	0	hsa-let-7d	-0.25	0.87
ERCC4	excision repair cross-complementing rodent repair deficiency, complementation group 4	1	1	0	0	0	0	0	0	hsa-let-7f	-0.23	0.95
ERCC6	excision repair cross-complementing rodent repair deficiency, complementation group 6	1	1	0	0	0	0	0	0	hsa-let-7d	-0.45	0.91
FAM103A1	family with sequence similarity 103, member A1	1	1	0	0	0	0	0	0	hsa-let-7d	-0.45	0.66
FAM118A	family with sequence similarity 118, member A	1	1	0	0	0	0	0	0	hsa-let-7d	-0.26	0.95
FAS	Fas (TNF receptor superfamily, member 6)	1	1	0	0	0	0	0	0	hsa-miR-98	-0.35	0.54
FBXL12	F-box and leucine-rich repeat protein 12	1	1	0	0	0	0	0	0	hsa-let-7a	-0.33	0.87
FBXO45	F-box protein 45	1	1	0	0	0	0	0	0	hsa-let-7a	-0.15	0.9
FGD6	FYVE, RhoGEF and PH domain containing 6	1	1	0	0	0	0	0	0	hsa-let-7a	-0.35	0.98
FLJ45032	similar to F40B5.2b	1	1	0	0	0	0	0	0	hsa-let-7f	-0.29	0.48
FNIP1	folliculin interacting protein 1	1	1	0	0	0	0	0	0	hsa-miR-98	-0.3	0.98
FRAS1	Fraser syndrome 1	1	1	0	0	0	0	0	0	hsa-let-7b	-0.35	0.97
FRMD4B	FERM domain containing 4B	1	1	0	0	0	0	0	0	hsa-let-7d	-0.38	0.82
FZD4	frizzled homolog 4 (Drosophila)	1	1	0	0	0	0	0	0	hsa-let-7f	-0.34	0.93
GABBR2	gamma-aminobutyric acid (GABA) B receptor, 2	1	1	0	0	0	0	0	0	hsa-let-7b	-0.1	0.7
GALE	UDP-galactose-4-epimerase	1	1	0	0	0	0	0	0	hsa-let-7a	-0.2	0.72
GAN	giant axonal neuropathy (gigaxonin)	1	1	0	0	0	0	0	0	hsa-let-7f	-0.33	0.96
GDAP2	ganglioside induced differentiation associated protein 2	1	1	0	0	1	0	1	0	hsa-let-7d	-0.58	0.75
GEMIN7	gem (nuclear organelle) associated protein 7	1	1	0	0	0	0	0	0	hsa-let-7f	-0.36	< 0.1
GIPC1	GIPC PDZ domain containing family, member 1	1	1	0	0	0	0	0	0	hsa-let-7g	-0.17	0.96
GJC1	gap junction protein, gamma 1, 45kDa	1	1	0	0	1	0	0	1	hsa-miR-98	-0.51	0.98
GLT8D3	glycosyltransferase 8 domain containing 3	1	1	0	0	1	0	0	1	hsa-let-7g	-0.47	0.99
GNPTAB	N-acetylglucosamine-1-phosphate transferase, alpha and beta subunits	1	1	0	0	0	0	0	0	hsa-let-7a	-0.45	0.98
GOLT1B	golgi transport 1 homolog B (S. cerevisiae)	1	1	0	0	0	0	0	0	hsa-let-7g	-0.27	0.97
HDHD1A	haloacid dehalogenase-like hydrolase domain containing 1A	1	1	0	0	0	0	0	0	hsa-let-7f	-0.3	0.21
HDLBP	high density lipoprotein binding protein	1	1	0	0	1	0	1	0	hsa-let-7d	-0.22	0.98
HIF1AN	hypoxia-inducible factor 1, alpha subunit inhibitor	1	1	0	0	1	0	1	0	hsa-let-7f	-0.45	0.62
HS2ST1	heparan sulfate 2-O-sulfotransferase 1	1	1	0	0	0	0	0	0	hsa-miR-98	-0.12	0.78
ICMT	isoprenylcysteine carboxyl methyltransferase	1	1	0	0	0	0	0	0	hsa-let-7f	-0.07	0.98
IGF2BP3	insulin-like growth factor 2 mRNA binding protein 3	1	1	0	0	0	0	0	0	hsa-let-7a	-0.38	0.95
IGSF1	immunoglobulin superfamily, member 1	1	1	0	0	0	0	0	0	hsa-let-7f	-0.36	0.86
INSR	insulin receptor	1	1	0	0	0	0	0	0	hsa-let-7i	-0.19	0.98
INTS2	integrator complex subunit 2	1	1	0	0	1	0	0	1	hsa-let-7a	-0.31	0.89
IPO9	importin 9	1	1	0	0	1	0	0	1	hsa-let-7d	-0.2	< 0.1
IRS2	insulin receptor substrate 2	1	1	0	0	0	0	0	0	hsa-let-7b	-0.25	0.97
KATNAL1	katanin p60 subunit A-like 1	1	1	0	0	1	0	1	0	hsa-let-7a	-0.31	0.97
KCTD17	potassium channel tetramerisation domain containing 17	1	1	0	0	0	0	0	0	hsa-let-7b	-0.19	0.96
KIAA1274	KIAA1274	1	1	0	0	0	0	0	0	hsa-miR-98	-0.33	0.98
KIAA1305	KIAA1305	1	1	0	0	2	0	2	0	hsa-let-7d	-0.38	0.98
KIAA1539	KIAA1539	1	1	0	0	0	0	0	0	hsa-let-7a	-0.19	0.97

Gene	Gene name	Conserved sites				Poorly conserved sites				Representative miRNA	Total context score	Aggregate PCT
		total	8mer	7mer-m8	7mer-1A	total	8merPC	7mer-m8	7mer-1A			
KIF21B	kinesin family member 21B	1	1	0	0	0	0	0	0	hsa-let-7d	0.02	0.98
KLHDC8B	kelch domain containing 8B	1	1	0	0	0	0	0	0	hsa-let-7a	-0.29	0.95
KLHL6	kelch-like 6 (Drosophila)	1	1	0	0	0	0	0	0	hsa-let-7d	-0.27	0.98
L2HGDH	L-2-hydroxyglutarate dehydrogenase	1	1	0	0	0	0	0	0	hsa-let-7a	-0.15	0.98
LAMP2	lysosomal-associated membrane protein 2	1	1	0	0	1	0	0	1	hsa-let-7d	-0.44	0.48
LBA1	lupus brain antigen 1	1	1	0	0	0	0	0	0	hsa-let-7b	-0.32	0.94
LBR	lamin B receptor	1	1	0	0	0	0	0	0	hsa-let-7d	-0.26	0.98
LIN28	lin-28 homolog (C. elegans)	1	1	0	0	0	0	0	0	hsa-let-7i	-0.33	0.98
LRIG1	leucine-rich repeats and immunoglobulin-like domains 1	1	1	0	0	0	0	0	0	hsa-let-7a	N/A	0.98
LRIG2	leucine-rich repeats and immunoglobulin-like domains 2	1	1	0	0	0	0	0	0	hsa-let-7d	-0.48	0.98
LRIG3	leucine-rich repeats and immunoglobulin-like domains 3	1	1	0	0	0	0	0	0	hsa-let-7a	-0.51	0.96
LSM11	LSM11, U7 small nuclear RNA associated	1	1	0	0	0	0	0	0	hsa-let-7b	-0.26	0.93
MAN2A2	mannosidase, alpha, class 2A, member 2	1	1	0	0	1	0	0	1	hsa-let-7f	-0.33	0.71
MAP3K1	mitogen-activated protein kinase kinase kinase 1	1	1	0	0	0	0	0	0	hsa-let-7a	-0.4	0.67
MAP4K3	mitogen-activated protein kinase kinase kinase kinase 3	1	1	0	0	0	0	0	0	hsa-miR-98	-0.51	0.96
MAP4K4	mitogen-activated protein kinase kinase kinase kinase 4	1	1	0	0	0	0	0	0	hsa-let-7a	-0.26	0.98
MAPK6	mitogen-activated protein kinase 6	1	1	0	0	0	0	0	0	hsa-let-7b	-0.41	0.89
MLL2	myeloid/lymphoid or mixed-lineage leukemia 2	1	1	0	0	0	0	0	0	hsa-let-7d	-0.23	0.98
MRS2	MRS2 magnesium homeostasis factor homolog (S. cerevisiae)	1	1	0	0	0	0	0	0	hsa-let-7f	-0.52	0.71
MXD1	MAX dimerization protein 1	1	1	0	0	0	0	0	0	hsa-let-7i	-0.29	0.98
MYCBP	c-myc binding protein	1	1	0	0	0	0	0	0	hsa-let-7d	-0.2	0.9
NAGA	N-acetylgalactosaminidase, alpha-	1	1	0	0	0	0	0	0	hsa-let-7a	-0.19	< 0.1
NDST2	N-deacetylase/N-sulfotransferase (heparan glucosaminyl) 2	1	1	0	0	0	0	0	0	hsa-let-7a	-0.25	0.93
NEK3	NIMA (never in mitosis gene a)-related kinase 3	1	1	0	0	0	0	0	0	hsa-let-7f	-0.33	0.84
NHLRC3	NHL repeat containing 3	1	1	0	0	0	0	0	0	hsa-let-7a	-0.36	0.94
NPHP3	nephronophthisis 3 (adolescent)	1	1	0	0	1	0	0	1	hsa-let-7g	-0.53	0.92
OSMR	oncostatin M receptor	1	1	0	0	0	0	0	0	hsa-let-7c	-0.46	0.79
PACS2	phosphofurin acidic cluster sorting protein 2	1	1	0	0	0	0	0	0	hsa-let-7a	-0.11	0.79
PCGF3	polycomb group ring finger 3	1	1	0	0	0	0	0	0	hsa-let-7a	-0.23	0.99
PDE12	phosphodiesterase 12	1	1	0	0	0	0	0	0	hsa-let-7a	-0.31	0.98
PDPR	pyruvate dehydrogenase phosphatase regulatory subunit	1	1	0	0	0	0	0	0	hsa-let-7d	-0.36	0.99
PEX11B	peroxisomal biogenesis factor 11B	1	1	0	0	0	0	0	0	hsa-let-7f	-0.34	0.17
PLXND1	plexin D1	1	1	0	0	0	0	0	0	hsa-let-7g	-0.29	0.97
POLL	polymerase (DNA directed), lambda	1	1	0	0	0	0	0	0	hsa-let-7b	-0.29	< 0.1
POLR2D	polymerase (RNA) II (DNA directed) polypeptide D	1	1	0	0	0	0	0	0	hsa-let-7b	-0.38	0.43
PPP1R15B	protein phosphatase 1, regulatory (inhibitor) subunit 15B	1	1	0	0	1	0	1	0	hsa-let-7c	-0.55	> 0.99
PQLC2	PQ loop repeat containing 2	1	1	0	0	0	0	0	0	hsa-let-7a	-0.24	0.95
PRPF38B	PRP38 pre-mRNA processing factor 38 (yeast) domain containing B	1	1	0	0	0	0	0	0	hsa-let-7d	-0.38	0.65
PRTG	protogenin homolog (Gallus gallus)	1	1	0	0	1	0	0	1	hsa-miR-98	-0.63	0.85
PTPRD	protein tyrosine phosphatase, receptor type, D	1	1	0	0	1	0	0	1	hsa-let-7a	-0.3	0.95
QARS	glutaminyl-tRNA synthetase	1	1	0	0	0	0	0	0	hsa-let-7i	-0.28	< 0.1
RANBP2	RAN binding protein 2	1	1	0	0	1	0	0	1	hsa-let-7g	-0.48	0.98
RDX	radixin	1	1	0	0	0	0	0	0	hsa-let-7a	-0.29	0.92
RICTOR	rapamycin-insensitive companion of mTOR	1	1	0	0	0	0	0	0	hsa-let-7g	-0.27	0.98
RRM2	ribonucleotide reductase M2 polypeptide	1	1	0	0	0	0	0	0	hsa-let-7a	-0.35	0.98
SCD	stearoyl-CoA desaturase (delta-9-desaturase)	1	1	0	0	0	0	0	0	hsa-miR-98	-0.31	0.98
SEMA4C	sema domain, immunoglobulin domain (Ig), transmembrane domain (TM) and short cyto	1	1	0	0	0	0	0	0	hsa-let-7i	-0.25	0.96
SEMA4F	sema domain, immunoglobulin domain (Ig), transmembrane domain (TM) and short cyto	1	1	0	0	0	0	0	0	hsa-let-7g	-0.38	0.79
SLC12A9	solute carrier family 12 (potassium/chloride transporters), member 9	1	1	0	0	0	0	0	0	hsa-let-7a	-0.24	0.96
SLC1A4	solute carrier family 1 (glutamate/neutral amino acid transporter), member 4	1	1	0	0	0	0	0	0	hsa-let-7d	-0.12	0.97
SLC2A12	solute carrier family 2 (facilitated glucose transporter), member 12	1	1	0	0	0	0	0	0	hsa-let-7f	-0.26	0.98
SLC35D2	solute carrier family 35, member D2	1	1	0	0	0	0	0	0	hsa-let-7d	-0.44	0.98
SMUG1	single-strand-selective monofunctional uracil-DNA glycosylase 1	1	1	0	0	0	0	0	0	hsa-let-7d	-0.33	0.84
SNX1	sorting nexin 1	1	1	0	0	0	0	0	0	hsa-let-7a	-0.16	0.12
SNX6	sorting nexin 6	1	1	0	0	0	0	0	0	hsa-let-7c	-0.33	0.92
STEAP3	STEAP family member 3	1	1	0	0	0	0	0	0	hsa-let-7d	-0.21	0.97
STX17	syntaxin 17	1	1	0	0	1	0	0	1	hsa-let-7g	-0.19	0.9
STX3	syntaxin 3	1	1	0	0	0	0	0	0	hsa-let-7g	-0.42	0.98
SYT11	synaptotagmin XI	1	1	0	0	0	0	0	0	hsa-let-7e	-0.13	0.97
TAF9B	TAF9B RNA polymerase II, TATA box binding protein (TBP)-associated factor, 31kDa	1	1	0	0	0	0	0	0	hsa-let-7a	-0.25	0.91
TBKBP1	TBK1 binding protein 1	1	1	0	0	0	0	0	0	hsa-let-7g	-0.37	0.93
TMEM194A	transmembrane protein 194A	1	1	0	0	0	0	0	0	hsa-let-7a	-0.18	0.82
TMEM2	transmembrane protein 2	1	1	0	0	0	0	0	0	hsa-let-7g	-0.38	0.98
TNFRSF1B	tumor necrosis factor receptor superfamily, member 1B	1	1	0	0	0	0	0	0	hsa-let-7c	-0.12	0.98
TP53	tumor protein p53	1	1	0	0	0	0	0	0	hsa-let-7d	-0.27	0.93
TRABD	TraB domain containing	1	1	0	0	0	0	0	0	hsa-let-7b	-0.18	< 0.1
TRAPPC1	trafficking protein particle complex 1	1	1	0	0	0	0	0	0	hsa-let-7a	N/A	< 0.1
TTC31	tetratricopeptide repeat domain 31	1	1	0	0	0	0	0	0	hsa-let-7d	-0.32	0.65
TTL	tubulin tyrosine ligase	1	1	0	0	0	0	0	0	hsa-let-7a	-0.15	0.96
TTLL4	tubulin tyrosine ligase-like family, member 4	1	1	0	0	0	0	0	0	hsa-let-7d	-0.25	0.93
UFM1	ubiquitin-fold modifier 1	1	1	0	0	0	0	0	0	hsa-let-7c	-0.34	0.56
UHRF2	ubiquitin-like, containing PHD and RING finger domains, 2	1	1	0	0	0	0	0	0	hsa-let-7d	-0.43	0.79
UTRN	utrophin	1	1	0	0	0	0	0	0	hsa-let-7a	-0.37	0.84
WDR37	WD repeat domain 37	1	1	0	0	0	0	0	0	hsa-let-7a	-0.23	0.98
XYLT1	xylosyltransferase I	1	1	0	0	0	0	0	0	hsa-let-7b	-0.03	0.99
ZC3H3	zinc finger CCCH-type containing 3	1	1	0	0	0	0	0	0	hsa-let-7d	-0.23	0.98
ZFYVE26	zinc finger, FYVE domain containing 26	1	1	0	0	0	0	0	0	hsa-let-7a	-0.39	0.98
ZNF280B	zinc finger protein 280B	1	1	0	0	1	0	1	0	hsa-let-7d	-0.33	0.93
ZNF322A	zinc finger protein 322A	1	1	0	0	0	0	0	0	hsa-let-7a	-0.46	0.85
ZNF473	zinc finger protein 473	1	1	0	0	0	0	0	0	hsa-let-7d	-0.29	0.95
ZNF566	zinc finger protein 566	1	1	0	0	0	0	0	0	hsa-let-7f	-0.42	< 0.1
ABL2	v-abl Abelson murine leukemia viral oncogene homolog 2 (arg, Abelson-related gene)	2	1	0	1	0	0	0	0	hsa-let-7d	-0.36	> 0.99
CBL	Cas-Br-M (murine) ecotropic retroviral transforming sequence	2	1	1	0	0	0	0	0	hsa-let-7f	-0.13	0.94
CDC34	cell division cycle 34 homolog (S. cerevisiae)	2	1	0	1	0	0	0	0	hsa-let-7d	-0.38	> 0.99
DTX2	deltex homolog 2 (Drosophila)	2	1	0	1	0	0	0	0	hsa-let-7d	-0.4	0.96
GALNT1	UDP-N-acetyl-alpha-D-galactosamine:polypeptide N-acetylgalactosaminyltransferase 1 ((	2	1	0	1	0	0	0	0	hsa-let-7a	-0.48	> 0.99
HIF3A	hypoxia inducible factor 3, alpha subunit	2	1	0	1	0	0	0	0	hsa-miR-98	-0.31	0.93
IGF2BP2	insulin-like growth factor 2 mRNA binding protein 2	2	1	0	1	0	0	0	0	hsa-let-7b	-0.37	> 0.99
NAP1L1	nucleosome assembly protein 1-like 1	2	1	0	1	0	0	0	0	hsa-let-7b	-0.44	> 0.99
RP5-1022P6.2	hypothetical protein KIAA1434	2	1	0	1	0	0	0	0	hsa-let-7f	-0.46	0.98
TGFBR1	transforming growth factor, beta receptor I (activin A receptor type II-like kinase, 53kDa)	2	1	0	1	0	0	0	0	hsa-let-7g	-0.57	> 0.99
ZNF200	zinc finger protein 200	2	1	0	1	0	0	0	0	hsa-let-7d	-0.4	0.98
ZNF275	zinc finger protein 275	2	1	1	0	0	0	0	0	hsa-let-7b	-0.49	0.98
CLCN5	chloride channel 5 (nephrolithiasis 2, X-linked, Dent disease)	3	1	1	1	3	0	0	3	hsa-let-7i	-0.58	> 0.99
IGF1R	insulin-like growth factor 1 receptor	3	1	1	1	0	0	0	0	hsa-let-7b	-0.56	> 0.99
TET3	tet oncogene family member 3	3	1	0	2	0	0	0	0	hsa-miR-98	-0.3	> 0.99
HMGA2	high mobility group AT-hook 2	6	1	3	2	1	0	0	1	hsa-let-7i	-1.35	> 0.99
DTX4	deltex 4 homolog (Drosophila)	1	0	0	1	1	1	0	0	hsa-let-7f	-0.17	0.96
SFRS12IP1	SFRS12-interacting protein 1	1	0	0	1	2	1	0	1	hsa-let-7g	-0.31	0.9

Gene	Gene name	Conserved sites				Poorly conserved sites				Representative miRNA	Total context score	Aggregate PCT
		total	8mer	7mer-m8	7mer-1A	total	8merPC	7mer-m8	7mer-1A			
WIPI2	WD repeat domain, phosphoinositide interacting 2	1	0	0	1	1	1	0	0	hsa-let-7e	-0.39	0.83
AAK1	AP2 associated kinase 1	1	0	1	0	1	0	0	1	hsa-let-7g	-0.08	0.9
ABCB9	ATP-binding cassette, sub-family B (MDR/TAP), member 9	1	0	1	0	0	0	0	0	hsa-miR-98	-0.26	0.94
ACTR10	actin-related protein 10 homolog (S. cerevisiae)	1	0	1	0	0	0	0	0	hsa-let-7f	-0.29	0.7
ACVR1B	activin A receptor, type IB	1	0	1	0	0	0	0	0	hsa-let-7g	-0.1	0.95
ADAMTS1	ADAM metallopeptidase with thrombospondin type 1 motif, 1	1	0	0	1	0	0	0	0	hsa-miR-98	-0.14	0.8
ADAMTS6	ADAM metallopeptidase with thrombospondin type 1 motif, 6	1	0	0	1	0	0	0	0	hsa-let-7d	-0.08	0.75
ADCY9	adenylate cyclase 9	1	0	0	1	0	0	0	0	hsa-let-7d	0.01	0.92
ADIPOR2	adiponectin receptor 2	1	0	0	1	0	0	0	0	hsa-let-7f	-0.18	0.91
ADRBK2	adrenergic, beta, receptor kinase 2	1	0	1	0	0	0	0	0	hsa-miR-98	-0.21	0.75
AFF2	AF4/FMR2 family, member 2	1	0	1	0	0	0	0	0	hsa-let-7a	-0.19	0.93
ALKBH1	alkB, alkylation repair homolog 1 (E. coli)	1	0	0	1	0	0	0	0	hsa-let-7d	-0.18	0.78
ALPK3	alpha-kinase 3	1	0	1	0	0	0	0	0	hsa-miR-98	-0.08	0.58
ANGPTL2	angiopoietin-like 2	1	0	0	1	0	0	0	0	hsa-let-7a	-0.1	0.93
ANKRD46	ankyrin repeat domain 46	1	0	1	0	0	0	0	0	hsa-let-7d	-0.19	0.78
ANKRD52	ankyrin repeat domain 52	1	0	0	1	3	0	2	1	hsa-let-7a	0	0.85
AP1S1	adaptor-related protein complex 1, sigma 1 subunit	1	0	1	0	0	0	0	0	hsa-let-7a	-0.1	0.76
ARHGAP20	Rho GTPase activating protein 20	1	0	1	0	0	0	0	0	hsa-let-7a	-0.14	0.92
ARHGEF15	Rho guanine nucleotide exchange factor (GEF) 15	1	0	1	0	0	0	0	0	hsa-let-7b	-0.19	0.93
ARID3A	AT rich interactive domain 3A (BRIGHT-like)	1	0	0	1	0	0	0	0	hsa-let-7f	-0.09	0.84
ARL5A	ADP-ribosylation factor-like 5A	1	0	0	1	0	0	0	0	hsa-let-7g	-0.17	0.93
ASAH3L	N-acylsphingosine amidohydrolase 3-like	1	0	1	0	0	0	0	0	hsa-let-7g	-0.18	0.91
ATG16L1	ATG16 autophagy related 16-like 1 (S. cerevisiae)	1	0	0	1	0	0	0	0	hsa-let-7a	-0.03	0.89
ATP2B4	ATPase, Ca++ transporting, plasma membrane 4	1	0	0	1	1	0	0	1	hsa-let-7f	-0.13	0.91
ATP7B	ATPase, Cu++ transporting, beta polypeptide	1	0	0	1	0	0	0	0	hsa-let-7b	-0.07	0.92
B3GAT3	beta-1,3-glucuronyltransferase 3 (glucuronosyltransferase I)	1	0	1	0	0	0	0	0	hsa-let-7a	-0.15	0.82
BACE2	beta-site APP-cleaving enzyme 2	1	0	1	0	0	0	0	0	hsa-let-7g	-0.29	0.9
BAHD1	bromo adjacent homology domain containing 1	1	0	0	1	0	0	0	0	hsa-let-7a	0.01	0.87
BCAP29	B-cell receptor-associated protein 29	1	0	0	1	0	0	0	0	hsa-let-7a	-0.21	0.92
BCL7A	B-cell CLL/lymphoma 7A	1	0	0	1	0	0	0	0	hsa-let-7f	-0.11	0.86
BIN3	bridging integrator 3	1	0	1	0	0	0	0	0	hsa-let-7g	-0.25	0.87
BNC2	basonuclin 2	1	0	0	1	0	0	0	0	hsa-let-7g	-0.11	0.84
BRD3	bromodomain containing 3	1	0	0	1	0	0	0	0	hsa-let-7a	-0.21	0.93
BTBD3	BTB (POZ) domain containing 3	1	0	0	1	0	0	0	0	hsa-let-7c	-0.23	0.93
BTBD9	BTB (POZ) domain containing 9	1	0	1	0	0	0	0	0	hsa-let-7a	-0.16	0.95
BTF3L4	basic transcription factor 3-like 4	1	0	1	0	0	0	0	0	hsa-let-7a	-0.12	0.88
BTG2	BTG family, member 2	1	0	0	1	0	0	0	0	hsa-let-7i	-0.08	0.89
BZW2	basic leucine zipper and W2 domains 2	1	0	1	0	0	0	0	0	hsa-let-7a	-0.25	0.77
C11orf57	chromosome 11 open reading frame 57	1	0	1	0	0	0	0	0	hsa-let-7f	-0.29	0.83
C12orf51	chromosome 12 open reading frame 51	1	0	1	0	0	0	0	0	hsa-let-7a	-0.08	0.9
C15orf29	chromosome 15 open reading frame 29	1	0	1	0	0	0	0	0	hsa-let-7b	-0.21	0.93
C16orf63	chromosome 16 open reading frame 63	1	0	0	1	0	0	0	0	hsa-let-7a	-0.13	0.86
C1orf26	chromosome 1 open reading frame 26	1	0	1	0	0	0	0	0	hsa-let-7b	-0.23	0.8
C3orf63	chromosome 3 open reading frame 63	1	0	0	1	0	0	0	0	hsa-let-7d	-0.1	0.88
C9orf7	chromosome 9 open reading frame 7	1	0	1	0	0	0	0	0	hsa-let-7a	-0.12	0.8
CALM1	calmodulin 1 (phosphorylase kinase, delta)	1	0	0	1	0	0	0	0	hsa-let-7f	-0.11	0.92
CALU	calumenin	1	0	0	1	0	0	0	0	hsa-let-7b	-0.02	0.79
CAP1	CAP, adenylate cyclase-associated protein 1 (yeast)	1	0	0	1	0	0	0	0	hsa-let-7b	-0.11	0.87
CAPN3	calpain 3, (p94)	1	0	0	1	0	0	0	0	hsa-let-7g	-0.13	0.91
CBX2	chromobox homolog 2 (Pc class homolog, Drosophila)	1	0	1	0	1	0	0	1	hsa-let-7b	-0.13	0.92
CCDC100	coiled-coil domain containing 100	1	0	1	0	0	0	0	0	hsa-let-7a	-0.23	0.93
CCNJL	cyclin J-like	1	0	0	1	0	0	0	0	hsa-let-7f	-0.11	0.86
CDC14B	CDC14 cell division cycle 14 homolog B (S. cerevisiae)	1	0	1	0	0	0	0	0	hsa-let-7b	-0.1	0.77
CDC25A	cell division cycle 25 homolog A (S. pombe)	1	0	1	0	2	0	1	1	hsa-let-7d	-0.36	0.71
CDC42SE1	CDC42 small effector 1	1	0	1	0	1	0	0	1	hsa-let-7d	-0.18	0.71
CDCA8	cell division cycle associated 8	1	0	1	0	0	0	0	0	hsa-let-7d	-0.18	0.83
CDYL	chromodomain protein, Y-like	1	0	0	1	0	0	0	0	hsa-let-7g	-0.1	0.69
CEP135	centrosomal protein 135kDa	1	0	1	0	0	0	0	0	hsa-let-7b	-0.38	0.95
CEP164	centrosomal protein 164kDa	1	0	0	1	0	0	0	0	hsa-let-7f	-0.06	0.92
CERCAM	cerebral endothelial cell adhesion molecule	1	0	1	0	0	0	0	0	hsa-let-7d	-0.14	0.87
CGNL1	cingulin-like 1	1	0	1	0	0	0	0	0	hsa-let-7d	-0.18	0.92
CHD4	chromodomain helicase DNA binding protein 4	1	0	1	0	0	0	0	0	hsa-let-7a	-0.16	0.78
CHD7	chromodomain helicase DNA binding protein 7	1	0	0	1	0	0	0	0	hsa-let-7a	-0.13	0.82
CLASP2	cytoplasmic linker associated protein 2	1	0	1	0	0	0	0	0	hsa-let-7g	-0.21	0.91
CNOT2	CCR4-NOT transcription complex, subunit 2	1	0	0	1	0	0	0	0	hsa-let-7d	-0.15	0.85
CNOT6L	CCR4-NOT transcription complex, subunit 6-like	1	0	0	1	0	0	0	0	hsa-miR-98	-0.07	0.9
COL1A1	collagen, type I, alpha 1	1	0	1	0	0	0	0	0	hsa-let-7d	-0.19	0.87
COL24A1	collagen, type XXIV, alpha 1	1	0	1	0	0	0	0	0	hsa-let-7a	-0.22	0.91
COL4A1	collagen, type IV, alpha 1	1	0	0	1	0	0	0	0	hsa-let-7a	-0.14	0.9
COL4A2	collagen, type IV, alpha 2	1	0	0	1	0	0	0	0	hsa-miR-98	-0.18	0.94
COL4A5	collagen, type IV, alpha 5 (Alport syndrome)	1	0	0	1	0	0	0	0	hsa-let-7a	-0.12	0.91
CPD	carboxypeptidase D	1	0	1	0	0	0	0	0	hsa-let-7d	-0.21	0.94
CPEB3	cytoplasmic polyadenylation element binding protein 3	1	0	0	1	1	0	0	1	hsa-let-7d	-0.24	0.94
CPEB4	cytoplasmic polyadenylation element binding protein 4	1	0	0	1	1	0	0	1	hsa-let-7d	-0.21	0.95
CPM	carboxypeptidase M	1	0	1	0	0	0	0	0	hsa-let-7a	-0.26	0.9
CPSF4	cleavage and polyadenylation specific factor 4, 30kDa	1	0	1	0	0	0	0	0	hsa-let-7a	-0.1	0.82
CREM	cAMP responsive element modulator	1	0	0	1	0	0	0	0	hsa-let-7i	-0.22	0.77
CROP	cisplatin resistance-associated overexpressed protein	1	0	0	1	0	0	0	0	hsa-let-7g	-0.04	0.82
CRTAP	cartilage associated protein	1	0	0	1	0	0	0	0	hsa-let-7d	-0.07	0.94
CRY2	cryptochrome 2 (photolyase-like)	1	0	1	0	0	0	0	0	hsa-let-7a	-0.1	0.89
CTHRC1	collagen triple helix repeat containing 1	1	0	1	0	0	0	0	0	hsa-let-7a	-0.27	0.76
CTNS	cystinosis, nephropathic	1	0	1	0	0	0	0	0	hsa-let-7d	-0.12	0.86
CTSC	cathepsin C	1	0	1	0	0	0	0	0	hsa-let-7d	-0.1	0.93
DAPK1	death-associated protein kinase 1	1	0	1	0	1	0	1	0	hsa-let-7g	-0.16	0.88
DHX57	DEAH (Asp-Glu-Ala-Asp/His) box polypeptide 57	1	0	1	0	0	0	0	0	hsa-let-7i	-0.26	0.66
DIAPH2	diaphanous homolog 2 (Drosophila)	1	0	1	0	1	0	1	0	hsa-let-7g	-0.54	0.97
DKK3	dickkopf homolog 3 (Xenopus laevis)	1	0	1	0	0	0	0	0	hsa-let-7d	-0.1	0.89
DLST	dihydrolipoamide S-succinyltransferase (E2 component of 2-oxo-glutarate complex)	1	0	1	0	0	0	0	0	hsa-let-7d	-0.2	0.94
DNAJC1	DnaJ (Hsp40) homolog, subfamily C, member 1	1	0	0	1	0	0	0	0	hsa-let-7d	-0.26	0.77
DNAL1	dynein, axonemal, light chain 1	1	0	1	0	0	0	0	0	hsa-let-7a	-0.1	0.9
DPF2	D4, zinc and double PHD fingers family 2	1	0	1	0	0	0	0	0	hsa-let-7g	-0.25	0.91
DPYSL3	dihydropyrimidinase-like 3	1	0	0	1	0	0	0	0	hsa-let-7a	-0.06	0.72
DUSP1	dual specificity phosphatase 1	1	0	0	1	0	0	0	0	hsa-let-7a	-0.21	0.82
DUSP16	dual specificity phosphatase 16	1	0	0	1	1	0	1	0	hsa-let-7d	-0.32	0.86
DUSP4	dual specificity phosphatase 4	1	0	0	1	0	0	0	0	hsa-let-7f	-0.19	0.94
DYRK1A	dual-specificity tyrosine-(Y)-phosphorylation regulated kinase 1A	1	0	1	0	0	0	0	0	hsa-let-7d	-0.19	0.79
DZIP1	DAZ interacting protein 1	1	0	0	1	0	0	0	0	hsa-let-7g	-0.18	0.94
E2F2	E2F transcription factor 2	1	0	1	0	1	0	1	0	hsa-let-7d	-0.25	0.94
EEA1	early endosome antigen 1	1	0	1	0	0	0	0	0	hsa-let-7a	-0.26	0.94

Gene	Gene name	Conserved sites				Poorly conserved sites				Representative miRNA	Total context score	Aggregate PCT
		total	8mer	7mer-m8	7mer-1A	total	8merPC	7mer-m8	7mer-1A			
EEF2K	eukaryotic elongation factor-2 kinase	1	0	1	0	0	0	0	0	hsa-let-7d	-0.25	0.95
EGLN2	egl nine homolog 2 (C. elegans)	1	0	0	1	0	0	0	0	hsa-let-7g	-0.09	0.9
EIF2C1	eukaryotic translation initiation factor 2C, 1	1	0	0	1	1	0	1	0	hsa-let-7a	-0.06	0.92
EIF2C3	eukaryotic translation initiation factor 2C, 3	1	0	0	1	0	0	0	0	hsa-let-7f	-0.13	0.93
EIF2C4	eukaryotic translation initiation factor 2C, 4	1	0	0	1	0	0	0	0	hsa-let-7f	-0.17	0.83
EIF4G2	eukaryotic translation initiation factor 4 gamma, 2	1	0	1	0	0	0	0	0	hsa-let-7d	-0.3	0.91
ELF4	E74-like factor 4 (ets domain transcription factor)	1	0	1	0	0	0	0	0	hsa-let-7a	-0.17	0.93
EPB41	erythrocyte membrane protein band 4.1 (elliptocytosis 1, RH-linked)	1	0	0	1	0	0	0	0	hsa-let-7a	-0.06	0.78
EPHA4	EPH receptor A4	1	0	0	1	0	0	0	0	hsa-let-7f	-0.18	0.83
ERO1L	ERO1-like (S. cerevisiae)	1	0	0	1	0	0	0	0	hsa-let-7a	-0.18	0.82
ETNK1	ethanolamine kinase 1	1	0	1	0	0	0	0	0	hsa-let-7b	-0.11	0.86
EZH1	enhancer of zeste homolog 1 (Drosophila)	1	0	1	0	0	0	0	0	hsa-let-7a	-0.15	0.83
EZH2	enhancer of zeste homolog 2 (Drosophila)	1	0	1	0	0	0	0	0	hsa-let-7a	N/A	0.79
FAM104A	family with sequence similarity 104, member A	1	0	1	0	0	0	0	0	hsa-let-7d	-0.24	0.89
FAM125B	family with sequence similarity 125, member B	1	0	0	1	0	0	0	0	hsa-let-7a	0.06	0.75
FAM135A	family with sequence similarity 135, member A	1	0	0	1	0	0	0	0	hsa-let-7f	-0.22	0.8
FAM160B2	family with sequence similarity 160, member B2	1	0	0	1	0	0	0	0	hsa-let-7a	0	0.91
FAM43A	family with sequence similarity 43, member A	1	0	0	1	1	0	1	0	hsa-let-7d	-0.06	0.72
FARP1	FERM, RhoGEF (ARHGEF) and pleckstrin domain protein 1 (chondrocyte-derived)	1	0	0	1	0	0	0	0	hsa-let-7f	-0.15	0.93
FBXL19	F-box and leucine-rich repeat protein 19	1	0	0	1	0	0	0	0	hsa-let-7f	-0.03	0.78
FOXP1	forkhead box P1	1	0	0	1	0	0	0	0	hsa-let-7g	-0.07	0.84
FRS2	fibroblast growth factor receptor substrate 2	1	0	0	1	0	0	0	0	hsa-let-7i	-0.14	0.81
GAB2	GRB2-associated binding protein 2	1	0	1	0	1	0	1	0	hsa-let-7b	-0.13	0.6
GABPA	GA binding protein transcription factor, alpha subunit 60kDa	1	0	0	1	0	0	0	0	hsa-let-7g	-0.12	0.87
GALC	galactosylceramidase	1	0	1	0	0	0	0	0	hsa-let-7f	-0.3	0.89
GALNT2	UDP-N-acetyl-alpha-D-galactosamine:polypeptide N-acetylgalactosaminyltransferase 2 (G	1	0	0	1	0	0	0	0	hsa-let-7g	-0.21	0.94
GALNTL2	UDP-N-acetyl-alpha-D-galactosamine:polypeptide N-acetylgalactosaminyltransferase-like	1	0	1	0	0	0	0	0	hsa-let-7a	-0.24	0.94
GGA3	golgi associated, gamma adaptin ear containing, ARF binding protein 3	1	0	1	0	0	0	0	0	hsa-let-7a	-0.14	0.91
GHR	growth hormone receptor	1	0	0	1	0	0	0	0	hsa-miR-98	-0.19	0.94
GLRX	glutaredoxin (thioltransferase)	1	0	1	0	0	0	0	0	hsa-let-7d	-0.24	0.76
GNG5	guanine nucleotide binding protein (G protein), gamma 5	1	0	1	0	0	0	0	0	hsa-let-7a	-0.19	0.74
GNS	glucosamine (N-acetyl)-6-sulfatase (Sanfilippo disease IIID)	1	0	0	1	0	0	0	0	hsa-let-7a	-0.02	0.85
GOLGA4	golgi autoantigen, golgin subfamily a, 4	1	0	0	1	0	0	0	0	hsa-let-7g	-0.2	0.87
GOLGA7	golgi autoantigen, golgin subfamily a, 7	1	0	1	0	0	0	0	0	hsa-let-7d	-0.12	0.64
GOPC	golgi associated PDZ and coiled-coil motif containing	1	0	0	1	0	0	0	0	hsa-let-7g	-0.05	0.78
GPATCH3	G patch domain containing 3	1	0	0	1	0	0	0	0	hsa-let-7d	-0.06	0.82
GPR137	G protein-coupled receptor 137	1	0	1	0	0	0	0	0	hsa-let-7b	-0.15	0.69
GREB1	GREB1 protein	1	0	1	0	0	0	0	0	hsa-let-7f	-0.2	0.66
GRPEL2	GrpE-like 2, mitochondrial (E. coli)	1	0	1	0	0	0	0	0	hsa-let-7i	-0.28	0.89
GTF2I	general transcription factor II, i	1	0	0	1	0	0	0	0	hsa-let-7d	-0.23	0.74
HABP4	hyaluronan binding protein 4	1	0	1	0	0	0	0	0	hsa-let-7g	-0.21	0.94
HIPK2	homeodomain interacting protein kinase 2	1	0	0	1	0	0	0	0	hsa-let-7d	-0.12	0.94
HK2	hexokinase 2	1	0	0	1	0	0	0	0	hsa-let-7a	-0.07	0.72
HMGA1	high mobility group AT-hook 1	1	0	1	0	0	0	0	0	hsa-let-7i	-0.3	0.92
HTR4	5-hydroxytryptamine (serotonin) receptor 4	1	0	1	0	0	0	0	0	hsa-let-7a	-0.12	0.93
IKBKAP	inhibitor of kappa light polypeptide gene enhancer in B-cells, kinase complex-associated p	1	0	1	0	0	0	0	0	hsa-let-7a	-0.27	0.85
IKBKE	inhibitor of kappa light polypeptide gene enhancer in B-cells, kinase epsilon	1	0	1	0	0	0	0	0	hsa-let-7b	-0.11	0.89
INPP5A	inositol polyphosphate-5-phosphatase, 40kDa	1	0	0	1	0	0	0	0	hsa-let-7d	-0.07	0.93
IQCB1	IQ motif containing B1	1	0	1	0	0	0	0	0	hsa-let-7d	-0.27	0.82
KCMF1	potassium channel modulatory factor 1	1	0	0	1	0	0	0	0	hsa-let-7g	-0.11	0.93
KCNC4	potassium voltage-gated channel, Shaw-related subfamily, member 4	1	0	1	0	0	0	0	0	hsa-let-7b	-0.07	0.72
KCTD10	potassium channel tetramerisation domain containing 10	1	0	1	0	0	0	0	0	hsa-let-7a	0.02	0.83
KIAA0329	KIAA0329	1	0	0	1	1	0	0	1	hsa-let-7d	-0.2	0.94
KIAA1147	KIAA1147	1	0	0	1	0	0	0	0	hsa-let-7f	-0.14	0.84
KIAA1467	KIAA1467	1	0	1	0	0	0	0	0	hsa-let-7a	-0.11	0.7
KIAA1549	KIAA1549	1	0	1	0	0	0	0	0	hsa-let-7a	-0.04	0.94
KLF9	Kruppel-like factor 9	1	0	1	0	0	0	0	0	hsa-let-7a	-0.23	0.88
KLHL24	kelch-like 24 (Drosophila)	1	0	0	1	1	0	0	1	hsa-let-7a	0	0.83
KPNA1	karyopherin alpha 1 (importin alpha 5)	1	0	0	1	1	0	0	1	hsa-let-7d	-0.17	0.91
KTELC1	KTEL (Lys-Tyr-Glu-Leu) containing 1	1	0	0	1	0	0	0	0	hsa-let-7f	-0.15	0.8
LBH	limb bud and heart development homolog (mouse)	1	0	0	1	0	0	0	0	hsa-let-7g	-0.1	0.93
LCOR	ligand dependent nuclear receptor corepressor	1	0	0	1	0	0	0	0	hsa-let-7f	-0.11	0.81
LCORL	ligand dependent nuclear receptor corepressor-like	1	0	1	0	0	0	0	0	hsa-let-7a	-0.22	0.95
LIMD1	LIM domains containing 1	1	0	0	1	0	0	0	0	hsa-let-7b	-0.13	0.87
LIMD2	LIM domain containing 2	1	0	1	0	3	0	3	0	hsa-let-7i	-0.45	0.94
LOC653319	hypothetical protein LOC653319	1	0	1	0	0	0	0	0	hsa-let-7i	-0.15	0.89
LOC90379	hypothetical protein BC002926	1	0	1	0	0	0	0	0	hsa-let-7b	-0.13	0.93
LPGAT1	lysophosphatidylglycerol acyltransferase 1	1	0	0	1	1	0	0	1	hsa-let-7g	-0.26	0.89
LRFN4	leucine rich repeat and fibronectin type III domain containing 4	1	0	0	1	0	0	0	0	hsa-let-7a	-0.06	0.79
LY75	lymphocyte antigen 75	1	0	0	1	0	0	0	0	hsa-let-7d	-0.26	0.8
LYPLA3	lysophospholipase 3 (lysosomal phospholipase A2)	1	0	1	0	0	0	0	0	hsa-let-7b	-0.07	0.86
MAP3K3	mitogen-activated protein kinase kinase kinase 3	1	0	0	1	1	0	0	1	hsa-let-7b	-0.01	0.78
MAP3K7IP2	mitogen-activated protein kinase kinase kinase 7 interacting protein 2	1	0	0	1	0	0	0	0	hsa-let-7b	-0.19	0.77
MAPK11	mitogen-activated protein kinase 11	1	0	1	0	0	0	0	0	hsa-let-7d	-0.09	0.72
MAPK1IP1L	mitogen-activated protein kinase 1 interacting protein 1-like	1	0	1	0	1	0	0	1	hsa-let-7d	-0.13	0.94
MDFI	MyoD family inhibitor	1	0	1	0	0	0	0	0	hsa-let-7g	-0.1	0.92
MECP2	methyl CpG binding protein 2 (Rett syndrome)	1	0	1	0	0	0	0	0	hsa-let-7b	-0.08	0.83
MED6	mediator complex subunit 6	1	0	0	1	0	0	0	0	hsa-let-7d	-0.13	0.71
MEF2D	myocyte enhancer factor 2D	1	0	1	0	1	0	1	0	hsa-let-7a	0	0.91
MEIS2	Meis homeobox 2	1	0	0	1	0	0	0	0	hsa-let-7d	-0.21	0.81
MEX3A	mex-3 homolog A (C. elegans)	1	0	0	1	0	0	0	0	hsa-miR-98	-0.19	0.91
MGA	MAX gene associated	1	0	0	1	0	0	0	0	hsa-let-7f	-0.11	0.87
MGAT4A	mannosyl (alpha-1,3-)-glycoprotein beta-1,4-N-acetylglucosaminyltransferase, isozyme A	1	0	1	0	0	0	0	0	hsa-let-7a	-0.29	0.95
MGLL	monoglyceride lipase	1	0	1	0	1	0	0	1	hsa-let-7i	-0.07	0.97
MLLT10	myeloid/lymphoid or mixed-lineage leukemia (trithorax homolog, Drosophila); translocat	1	0	0	1	0	0	0	0	hsa-let-7i	-0.2	0.85
MLLT4	myeloid/lymphoid or mixed-lineage leukemia (trithorax homolog, Drosophila); translocat	1	0	1	0	0	0	0	0	hsa-let-7c	-0.12	0.86
MNT	MAX binding protein	1	0	0	1	0	0	0	0	hsa-let-7d	-0.01	0.88
MOBKL3	MOB1, Mps One Binder kinase activator-like 3 (yeast)	1	0	0	1	0	0	0	0	hsa-let-7a	-0.21	0.86
MON2	MON2 homolog (S. cerevisiae)	1	0	0	1	0	0	0	0	hsa-let-7d	-0.15	0.94
MTMR12	myotubularin related protein 12	1	0	0	1	0	0	0	0	hsa-let-7a	-0.09	0.87
MTMR3	myotubularin related protein 3	1	0	1	0	0	0	0	0	hsa-let-7a	-0.13	0.79
MTPN	myotrophin	1	0	0	1	0	0	0	0	hsa-let-7d	-0.13	0.74
MTUS1	mitochondrial tumor suppressor 1	1	0	1	0	0	0	0	0	hsa-let-7d	-0.2	0.71
MUTED	muted homolog (mouse)	1	0	0	1	0	0	0	0	hsa-let-7c	-0.15	0.75
MYRIP	myosin VIIA and Rab interacting protein	1	0	0	1	0	0	0	0	hsa-let-7d	-0.06	0.92
NAPEPLD	N-acyl phosphatidylethanolamine phospholipase D	1	0	1	0	0	0	0	0	hsa-let-7a	-0.08	0.94
NAT12	N-acetyltransferase 12	1	0	0	1	1	0	1	0	hsa-let-7a	-0.37	0.91
NID2	nidogen 2 (osteonidogen)	1	0	0	1	0	0	0	0	hsa-let-7g	-0.17	0.91
NIPA1	non imprinted in Prader-Willi/Angelman syndrome 1	1	0	1	0	1	0	0	1	hsa-let-7a	-0.33	0.95

Gene	Gene name	Conserved sites				Poorly conserved sites				Representative miRNA	Total context score	Aggregate PCT
		total	8mer	7mer-m8	7mer-1A	total	8merPC	7mer-m8	7mer-1A			
NKIRAS2	NFKB inhibitor interacting Ras-like 2	1	0	0	1	0	0	0	0	hsa-let-7a	-0.03	0.8
NLK	nemo-like kinase	1	0	1	0	0	0	0	0	hsa-let-7a	-0.14	0.91
NME4	non-metastatic cells 4, protein expressed in	1	0	1	0	0	0	0	0	hsa-let-7d	-0.18	0.91
NME6	non-metastatic cells 6, protein expressed in (nucleoside-diphosphate kinase)	1	0	1	0	0	0	0	0	hsa-let-7d	-0.25	0.94
NNT	nicotinamide nucleotide transhydrogenase	1	0	1	0	0	0	0	0	hsa-let-7d	-0.24	0.83
NOVA1	neuro-oncological ventral antigen 1	1	0	1	0	0	0	0	0	hsa-let-7d	-0.13	0.89
NPEPL1	aminopeptidase-like 1	1	0	0	1	0	0	0	0	hsa-let-7f	-0.14	0.94
NRAS	neuroblastoma RAS viral (v-ras) oncogene homolog	1	0	1	0	0	0	0	0	hsa-let-7f	-0.25	0.9
NRK	Nik related kinase	1	0	0	1	1	0	0	1	hsa-let-7i	-0.2	0.81
NUMBL	numb homolog (Drosophila)-like	1	0	0	1	0	0	0	0	hsa-let-7d	-0.01	0.94
OSBPL3	oxysterol binding protein-like 3	1	0	1	0	0	0	0	0	hsa-let-7a	-0.25	0.95
OTUD3	OTU domain containing 3	1	0	0	1	0	0	0	0	hsa-let-7b	-0.02	0.81
P4HA2	procollagen-proline, 2-oxoglutarate 4-dioxygenase (proline 4-hydroxylase), alpha polype	1	0	0	1	0	0	0	0	hsa-let-7d	-0.12	0.86
PAK1	p21 protein (Cdc42/Rac)-activated kinase 1	1	0	1	0	0	0	0	0	hsa-let-7d	-0.15	0.7
PANX2	pannexin 2	1	0	0	1	0	0	0	0	hsa-let-7b	-0.06	0.91
PDGFB	platelet-derived growth factor beta polypeptide (simian sarcoma viral (v-sis) oncogene hc	1	0	1	0	0	0	0	0	hsa-let-7f	-0.13	0.66
PDP2	pyruvate dehydrogenase phosphatase isoenzyme 2	1	0	1	0	0	0	0	0	hsa-let-7b	-0.27	0.69
PGRMC1	progesterone receptor membrane component 1	1	0	1	0	1	0	0	1	hsa-let-7d	-0.48	0.94
PHACTR2	phosphatase and actin regulator 2	1	0	0	1	0	0	0	0	hsa-let-7g	-0.18	0.93
PHC3	polyhomeotic homolog 3 (Drosophila)	1	0	1	0	1	0	1	0	hsa-let-7b	-0.2	0.74
PHF8	PHD finger protein 8	1	0	1	0	0	0	0	0	hsa-let-7d	-0.01	0.88
PIGA	phosphatidylinositol glycan anchor biosynthesis, class A (paroxysmal nocturnal hemoglob	1	0	1	0	0	0	0	0	hsa-let-7a	-0.29	0.95
PIK3IP1	phosphoinositide-3-kinase interacting protein 1	1	0	0	1	0	0	0	0	hsa-let-7a	-0.24	0.77
PKN2	protein kinase N2	1	0	0	1	1	0	0	1	hsa-let-7b	-0.18	0.97
PLAGL2	pleiomorphic adenoma gene-like 2	1	0	1	0	0	0	0	0	hsa-let-7b	-0.22	0.92
PLD3	phospholipase D family, member 3	1	0	1	0	0	0	0	0	hsa-let-7a	-0.1	0.82
PLDN	pallidin homolog (mouse)	1	0	1	0	0	0	0	0	hsa-let-7f	-0.15	0.94
PLEKHO1	pleckstrin homology domain containing, family O member 1	1	0	0	1	0	0	0	0	hsa-let-7d	-0.23	0.76
PNKD	paroxysmal nonkinesigenic dyskinesia	1	0	1	0	0	0	0	0	hsa-let-7a	-0.07	0.61
POGZ	pogo transposable element with ZNF domain	1	0	0	1	0	0	0	0	hsa-let-7a	-0.16	0.86
POLR3D	polymerase (RNA) III (DNA directed) polypeptide D, 44kDa	1	0	1	0	1	0	0	1	hsa-let-7d	-0.21	0.88
POU2F2	POU class 2 homeobox 2	1	0	0	1	1	0	1	0	hsa-let-7b	-0.22	0.87
PPARA	peroxisome proliferator-activated receptor alpha	1	0	0	1	1	0	0	1	hsa-let-7e	-0.06	0.92
PPP1R12B	protein phosphatase 1, regulatory (inhibitor) subunit 12B	1	0	1	0	0	0	0	0	hsa-let-7f	-0.18	0.77
PPP1R16B	protein phosphatase 1, regulatory (inhibitor) subunit 16B	1	0	1	0	0	0	0	0	hsa-let-7g	-0.12	0.94
PPTC7	PTC7 protein phosphatase homolog (S. cerevisiae)	1	0	0	1	0	0	0	0	hsa-let-7d	-0.11	0.76
PRDM1	PR domain containing 1, with ZNF domain	1	0	0	1	0	0	0	0	hsa-let-7a	-0.11	0.82
PSCD3	pleckstrin homology, Sec7 and coiled-coil domains 3	1	0	0	1	0	0	0	0	hsa-miR-98	-0.06	0.76
PTAR1	protein prenyltransferase alpha subunit repeat containing 1	1	0	0	1	2	0	0	2	hsa-let-7f	-0.34	0.96
PTPRU	protein tyrosine phosphatase, receptor type, U	1	0	1	0	0	0	0	0	hsa-let-7a	-0.12	0.86
PYGO2	pygopus homolog 2 (Drosophila)	1	0	0	1	0	0	0	0	hsa-let-7d	-0.09	0.86
RAB15	RAB15, member RAS onocogene family	1	0	1	0	2	0	2	0	hsa-let-7d	-0.18	0.87
RAB22A	RAB22A, member RAS oncogene family	1	0	1	0	0	0	0	0	hsa-let-7a	-0.21	0.77
RAB40C	RAB40C, member RAS oncogene family	1	0	1	0	0	0	0	0	hsa-let-7a	-0.1	0.85
RALB	v-ral simian leukemia viral oncogene homolog B (ras related; GTP binding protein)	1	0	1	0	0	0	0	0	hsa-let-7a	-0.15	0.94
RALGPS1	Ral GEF with PH domain and SH3 binding motif 1	1	0	0	1	0	0	0	0	hsa-let-7g	-0.14	0.94
RB1	retinoblastoma 1 (including osteosarcoma)	1	0	0	1	0	0	0	0	hsa-let-7a	-0.14	0.75
RBM9	RNA binding motif protein 9	1	0	0	1	0	0	0	0	hsa-miR-98	-0.24	0.93
RCN1	reticulocalbin 1, EF-hand calcium binding domain	1	0	0	1	0	0	0	0	hsa-let-7d	-0.2	0.85
RDH10	retinol dehydrogenase 10 (all-trans)	1	0	1	0	0	0	0	0	hsa-miR-98	-0.32	0.95
RIOK3	RIO kinase 3 (yeast)	1	0	1	0	0	0	0	0	hsa-let-7f	-0.28	0.94
RNF38	ring finger protein 38	1	0	0	1	0	0	0	0	hsa-let-7g	-0.16	0.83
RNF44	ring finger protein 44	1	0	0	1	0	0	0	0	hsa-let-7i	-0.17	0.94
RNF5	ring finger protein 5	1	0	1	0	0	0	0	0	hsa-let-7d	-0.23	0.78
RNF7	ring finger protein 7	1	0	1	0	0	0	0	0	hsa-let-7a	-0.13	0.92
RPS6KA3	ribosomal protein S6 kinase, 90kDa, polypeptide 3	1	0	1	0	1	0	0	1	hsa-miR-98	-0.19	0.94
RPUSD3	RNA pseudouridylate synthase domain containing 3	1	0	1	0	0	0	0	0	hsa-let-7g	-0.1	0.75
RRP1B	ribosomal RNA processing 1 homolog B (S. cerevisiae)	1	0	1	0	0	0	0	0	hsa-let-7d	-0.09	0.87
RUFY3	RUN and FYVE domain containing 3	1	0	1	0	0	0	0	0	hsa-let-7f	-0.16	0.93
SAMD12	sterile alpha motif domain containing 12	1	0	1	0	1	0	0	1	hsa-miR-98	-0.16	0.94
SCN5A	sodium channel, voltage-gated, type V, alpha subunit	1	0	1	0	0	0	0	0	hsa-let-7d	-0.04	0.94
SCUBE3	signal peptide, CUB domain, EGF-like 3	1	0	1	0	0	0	0	0	hsa-let-7i	0.01	0.79
SCYL3	SCY1-like 3 (S. cerevisiae)	1	0	0	1	0	0	0	0	hsa-let-7f	-0.21	0.88
SEC14L1	SEC14-like 1 (S. cerevisiae)	1	0	0	1	0	0	0	0	hsa-let-7g	-0.21	0.8
SEC24C	SEC24 related gene family, member C (S. cerevisiae)	1	0	0	1	0	0	0	0	hsa-miR-98	-0.11	0.79
SEC31B	SEC31 homolog B (S. cerevisiae)	1	0	1	0	0	0	0	0	hsa-let-7a	-0.15	0.6
SENP2	SUMO1/sentrin/SMT3 specific peptidase 2	1	0	0	1	0	0	0	0	hsa-let-7a	-0.17	0.8
SENP5	SUMO1/sentrin specific peptidase 5	1	0	0	1	0	0	0	0	hsa-let-7b	-0.18	0.94
SFRS12	splicing factor, arginine/serine-rich 12	1	0	0	1	0	0	0	0	hsa-let-7a	-0.08	0.82
SH2B3	SH2B adaptor protein 3	1	0	0	1	0	0	0	0	hsa-let-7g	-0.18	0.94
SH3RF3	SH3 domain containing ring finger 3	1	0	0	1	0	0	0	0	hsa-let-7b	-0.06	0.91
SLC25A24	solute carrier family 25 (mitochondrial carrier; phosphate carrier), member 24	1	0	1	0	0	0	0	0	hsa-let-7a	-0.24	0.91
SLC30A4	solute carrier family 30 (zinc transporter), member 4	1	0	0	1	0	0	0	0	hsa-let-7a	-0.15	0.94
SLC30A7	solute carrier family 30 (zinc transporter), member 7	1	0	1	0	0	0	0	0	hsa-let-7d	-0.2	0.86
SLC31A1	solute carrier family 31 (copper transporters), member 1	1	0	1	0	0	0	0	0	hsa-let-7g	-0.21	0.95
SLC45A4	solute carrier family 45, member 4	1	0	1	0	0	0	0	0	hsa-let-7d	-0.08	0.66
SLC5A6	solute carrier family 5 (sodium-dependent vitamin transporter), member 6	1	0	1	0	0	0	0	0	hsa-let-7f	-0.22	0.91
SMAD2	SMAD family member 2	1	0	1	0	0	0	0	0	hsa-let-7b	-0.15	0.87
SMAP2	stromal membrane-associated GTPase-activating protein 2	1	0	1	0	0	0	0	0	hsa-let-7c	-0.19	0.75
SMARCAD1	SWI/SNF-related, matrix-associated actin-dependent regulator of chromatin, subfamily a	1	0	1	0	1	0	1	0	hsa-let-7f	-0.62	0.95
SMARCC1	SWI/SNF related, matrix associated, actin dependent regulator of chromatin, subfamily c	1	0	1	0	0	0	0	0	hsa-let-7g	-0.2	0.9
SMCR7L	Smith-Magenis syndrome chromosome region, candidate 7-like	1	0	1	0	1	0	0	1	hsa-let-7d	-0.19	0.94
SMCR8	Smith-Magenis syndrome chromosome region, candidate 8	1	0	1	0	1	0	0	1	hsa-let-7d	-0.22	0.94
SNAP23	synaptosomal-associated protein, 23kDa	1	0	0	1	1	0	0	1	hsa-let-7a	-0.27	0.87
SNX16	sorting nexin 16	1	0	1	0	0	0	0	0	hsa-let-7f	-0.24	0.94
SNX30	sorting nexin family member 30	1	0	1	0	2	0	0	2	hsa-let-7d	-0.34	0.94
SOX13	SRY (sex determining region Y)-box 13	1	0	1	0	0	0	0	0	hsa-let-7g	-0.2	0.82
SPATA2	spermatogenesis associated 2	1	0	1	0	0	0	0	0	hsa-let-7a	-0.12	0.89
SPIRE1	spire homolog 1 (Drosophila)	1	0	0	1	0	0	0	0	hsa-let-7a	-0.12	0.9
SPTBN4	spectrin, beta, non-erythrocytic 4	1	0	0	1	0	0	0	0	hsa-let-7a	-0.01	0.84
SRGAP3	SLIT-ROBO Rho GTPase activating protein 3	1	0	0	1	1	0	1	0	hsa-miR-98	-0.19	0.94
SSH1	slingshot homolog 1 (Drosophila)	1	0	1	0	0	0	0	0	hsa-let-7a	-0.19	0.94
STARD3NL	STARD3 N-terminal like	1	0	0	1	0	0	0	0	hsa-let-7a	-0.15	0.9
STK24	serine/threonine kinase 24 (STE20 homolog, yeast)	1	0	0	1	0	0	0	0	hsa-let-7b	-0.15	0.89
STK40	serine/threonine kinase 40	1	0	1	0	0	0	0	0	hsa-let-7d	-0.27	0.95
STRBP	spermatid perinuclear RNA binding protein	1	0	0	1	0	0	0	0	hsa-let-7g	-0.17	0.85
STXBP5	syntaxin binding protein 5 (tomosyn)	1	0	0	1	0	0	0	0	hsa-let-7d	-0.16	0.72
SUCLG2	succinate-CoA ligase, GDP-forming, beta subunit	1	0	0	1	0	0	0	0	hsa-let-7a	-0.17	0.73
SURF4	surfeit 4	1	0	0	1	0	0	0	0	hsa-let-7a	-0.03	0.84

Gene	Gene name	Conserved sites				Poorly conserved sites				Representative miRNA	Total context score	Aggregate PCT
		total	8mer	7mer-m8	7mer-1A	total	8merPC	7mer-m8	7mer-1A			
SYT1	synaptotagmin I	1	0	0	1	0	0	0	0	hsa-let-7f	-0.17	0.83
TARBP2	TAR (HIV-1) RNA binding protein 2	1	0	1	0	0	0	0	0	hsa-let-7d	-0.19	0.91
TBX5	T-box 5	1	0	1	0	0	0	0	0	hsa-let-7g	-0.13	0.93
TEAD3	TEA domain family member 3	1	0	1	0	0	0	0	0	hsa-let-7a	-0.07	0.85
TEX261	testis expressed 261	1	0	0	1	1	0	1	0	hsa-let-7d	-0.07	0.73
THBS1	thrombospondin 1	1	0	0	1	0	0	0	0	hsa-let-7c	-0.25	0.83
THOC2	THO complex 2	1	0	0	1	0	0	0	0	hsa-let-7b	-0.22	0.8
TIMM17B	translocase of inner mitochondrial membrane 17 homolog B (yeast)	1	0	1	0	0	0	0	0	hsa-let-7f	-0.12	0.71
TMC7	transmembrane channel-like 7	1	0	0	1	0	0	0	0	hsa-let-7d	-0.25	0.84
TMED5	transmembrane emp24 protein transport domain containing 5	1	0	1	0	0	0	0	0	hsa-let-7f	-0.22	0.95
TMEM110	transmembrane protein 110	1	0	1	0	0	0	0	0	hsa-let-7d	-0.19	0.69
TMEM143	transmembrane protein 143	1	0	1	0	1	0	1	0	hsa-let-7a	-0.23	0.89
TMEM65	transmembrane protein 65	1	0	0	1	0	0	0	0	hsa-let-7g	-0.26	0.94
TNFAIP3	tumor necrosis factor, alpha-induced protein 3	1	0	1	0	0	0	0	0	hsa-let-7d	-0.08	0.88
TPP1	tripeptidyl peptidase I	1	0	1	0	1	0	0	1	hsa-miR-98	-0.25	0.96
TRIB1	tribbles homolog 1 (Drosophila)	1	0	0	1	0	0	0	0	hsa-let-7d	-0.11	0.93
TRIB2	tribbles homolog 2 (Drosophila)	1	0	0	1	0	0	0	0	hsa-let-7d	-0.09	0.85
TRIOBP	TRIO and F-actin binding protein	1	0	1	0	0	0	0	0	hsa-let-7a	-0.04	0.78
TSC1	tuberous sclerosis 1	1	0	1	0	0	0	0	0	hsa-let-7a	-0.16	0.94
TSC22D2	TSC22 domain family, member 2	1	0	0	1	0	0	0	0	hsa-let-7b	-0.17	0.83
TUSC2	tumor suppressor candidate 2	1	0	1	0	0	0	0	0	hsa-let-7d	-0.12	0.94
TXLNA	taxilin alpha	1	0	1	0	0	0	0	0	hsa-let-7b	-0.15	0.9
UBE2G2	ubiquitin-conjugating enzyme E2G 2 (UBC7 homolog, yeast)	1	0	1	0	0	0	0	0	hsa-let-7a	-0.13	0.73
UBFD1	ubiquitin family domain containing 1	1	0	0	1	0	0	0	0	hsa-let-7i	-0.07	0.74
UGCGL1	UDP-glucose ceramide glucosyltransferase-like 1	1	0	0	1	1	0	0	1	hsa-let-7d	-0.15	0.96
UHRF1	ubiquitin-like, containing PHD and RING finger domains, 1	1	0	0	1	0	0	0	0	hsa-let-7d	-0.2	0.92
ULK2	unc-51-like kinase 2 (C. elegans)	1	0	1	0	0	0	0	0	hsa-miR-98	-0.32	0.86
USP21	ubiquitin specific peptidase 21	1	0	0	1	0	0	0	0	hsa-let-7d	-0.08	0.91
USP24	ubiquitin specific peptidase 24	1	0	1	0	0	0	0	0	hsa-let-7e	-0.22	0.92
USP32	ubiquitin specific peptidase 32	1	0	1	0	0	0	0	0	hsa-let-7a	-0.23	0.94
USP38	ubiquitin specific peptidase 38	1	0	1	0	0	0	0	0	hsa-let-7g	-0.36	0.81
USP47	ubiquitin specific peptidase 47	1	0	0	1	0	0	0	0	hsa-let-7d	-0.18	0.94
USP6	ubiquitin specific peptidase 6 (Tre-2 oncogene)	1	0	1	0	0	0	0	0	hsa-let-7a	-0.2	0.94
UTP15	UTP15, U3 small nucleolar ribonucleoprotein, homolog (S. cerevisiae)	1	0	1	0	0	0	0	0	hsa-let-7b	-0.19	0.63
VASH2	vasohibin 2	1	0	0	1	0	0	0	0	hsa-let-7d	-0.16	0.93
VAV3	vav 3 guanine nucleotide exchange factor	1	0	0	1	0	0	0	0	hsa-let-7a	-0.14	0.9
VPS26B	vacuolar protein sorting 26 homolog B (S. pombe)	1	0	1	0	0	0	0	0	hsa-let-7a	-0.13	0.9
WASL	Wiskott-Aldrich syndrome-like	1	0	0	1	0	0	0	0	hsa-let-7d	-0.2	0.81
YAF2	YY1 associated factor 2	1	0	0	1	0	0	0	0	hsa-let-7a	-0.16	0.92
YBX2	Y box binding protein 2	1	0	1	0	0	0	0	0	hsa-let-7d	-0.1	0.57
YPEL2	yippee-like 2 (Drosophila)	1	0	0	1	1	0	0	1	hsa-let-7g	-0.17	0.92
YTHDF3	YTH domain family, member 3	1	0	0	1	0	0	0	0	hsa-let-7g	-0.15	0.83
ZBTB5	zinc finger and BTB domain containing 5	1	0	1	0	0	0	0	0	hsa-let-7f	-0.25	0.89
ZFYVE16	zinc finger, FYVE domain containing 16	1	0	1	0	0	0	0	0	hsa-let-7d	-0.17	0.58
ZNF282	zinc finger protein 282	1	0	1	0	0	0	0	0	hsa-let-7b	0.05	0.85
ZNF294	zinc finger protein 294	1	0	1	0	0	0	0	0	hsa-let-7d	-0.29	0.82
ZNF354A	zinc finger protein 354A	1	0	1	0	0	0	0	0	hsa-let-7d	-0.27	0.83
ZNF516	zinc finger protein 516	1	0	1	0	0	0	0	0	hsa-let-7a	-0.23	0.94
ZNF644	zinc finger protein 644	1	0	1	0	1	0	0	1	hsa-let-7d	-0.29	0.96
ZNF697	zinc finger protein 697	1	0	1	0	0	0	0	0	hsa-let-7g	-0.21	0.87
ZNF740	zinc finger protein 740	1	0	1	0	0	0	0	0	hsa-let-7d	-0.03	0.92
ACVR2A	activin A receptor, type IIA	2	0	0	2	0	0	0	0	hsa-let-7a	-0.3	0.97
BZW1	basic leucine zipper and W2 domains 1	2	0	2	0	0	0	0	0	hsa-let-7f	-0.5	> 0.99
C20orf194	chromosome 20 open reading frame 194	2	0	0	2	0	0	0	0	hsa-let-7a	-0.25	> 0.99
C3orf64	chromosome 3 open reading frame 64	2	0	1	1	0	0	0	0	hsa-let-7d	-0.26	> 0.99
CCND1	cyclin D1	2	0	0	2	0	0	0	0	hsa-let-7b	-0.1	0.98
CHST3	carbohydrate (chondroitin 6) sulfotransferase 3	2	0	1	1	0	0	0	0	hsa-let-7d	-0.13	0.98
CPEB2	cytoplasmic polyadenylation element binding protein 2	2	0	0	2	1	0	1	0	hsa-let-7d	-0.39	0.98
DICER1	dicer 1, ribonuclease type III	2	0	0	2	0	0	0	0	hsa-miR-98	-0.19	0.99
DLC1	deleted in liver cancer 1	2	0	0	2	0	0	0	0	hsa-let-7a	-0.31	> 0.99
FAM178A	family with sequence similarity 178, member A	2	0	2	0	0	0	0	0	hsa-miR-98	-0.41	0.96
FNDC3A	fibronectin type III domain containing 3A	2	0	1	1	0	0	0	0	hsa-let-7f	-0.47	0.96
FNDC3B	fibronectin type III domain containing 3B	2	0	0	2	0	0	0	0	hsa-let-7c	-0.2	0.96
ITGB3	integrin, beta 3 (platelet glycoprotein IIIa, antigen CD61)	2	0	1	1	0	0	0	0	hsa-let-7d	-0.23	> 0.99
MIB1	mindbomb homolog 1 (Drosophila)	2	0	2	0	0	0	0	0	hsa-let-7f	-0.31	0.99
PBX2	pre-B-cell leukemia homeobox 2	2	0	1	1	0	0	0	0	hsa-miR-98	-0.21	> 0.99
PBX3	pre-B-cell leukemia homeobox 3	2	0	1	1	0	0	0	0	hsa-miR-98	-0.39	0.99
PGM2L1	phosphoglucomutase 2-like 1	2	0	1	1	0	0	0	0	hsa-let-7d	-0.11	0.98
PLXNA4	plexin A4	2	0	1	1	0	0	0	0	hsa-let-7a	-0.19	> 0.99
PXDN	peroxidasin homolog (Drosophila)	2	0	0	2	0	0	0	0	hsa-let-7f	-0.23	> 0.99
STARD13	StAR-related lipid transfer (START) domain containing 13	2	0	0	2	0	0	0	0	hsa-let-7g	-0.5	> 0.99
ZNF362	zinc finger protein 362	2	0	2	0	0	0	0	0	hsa-let-7b	-0.1	0.84
CCND2	cyclin D2	3	0	0	3	0	0	0	0	hsa-let-7f	-0.18	> 0.99
ZNF512B	zinc finger protein 512B	3	0	3	0	0	0	0	0	hsa-let-7d	-0.34	> 0.99
ARID3B	AT rich interactive domain 3B (BRIGHT-like)	5	0	1	4	0	0	0	0	hsa-let-7i	-0.72	> 0.99
